# Dechdigliotoxins A–C, Three Novel Disulfide-Bridged Gliotoxin Dimers from Deep-Sea Sediment Derived Fungus *Dichotomomyces cejpii*

**DOI:** 10.3390/md17110596

**Published:** 2019-10-23

**Authors:** Zhaoming Liu, Zhen Fan, Zhanghua Sun, Hongxin Liu, Weimin Zhang

**Affiliations:** State Key Laboratory of Applied Microbiology Southern China, Guangdong Provincial Key Laboratory of Microbial Culture Collection and Application, Guangdong Open Laboratory of Applied Microbiology, Guangdong Institute of Microbiology, Guangdong Academy of Sciences, 100 Central Xianlie Road, Yuexiu District, Guangzhou 510070, China; liuzm@gdim.cn (Z.L.); zfan@sibs.ac.cn (Z.F.); sunzh@gzucm.edu.cn (Z.S.)

**Keywords:** dechdigliotoxins, disulfide-bridged, dimer, deep-sea derived fungus, quantum chemical calculations, cytotoxicity

## Abstract

Dechdigliotoxins A–C (**1–3**), which represented the first examples of gliotoxin dimers with an unprecedented exocyclic disulfide linkage, were obtained from a deep-sea derived fungus *Dichotomomyces cejpii* FS110. The structures of these compounds were elucidated on the basis of spectroscopic analysis and the absolute configurations were unambiguously determined through quantum chemical calculations, as well as DP4+ probability simulations. The proposed biosynthetic pathway suggested **1–3** were generated from unusual L-Phe and D-Ser. All the isolates were evaluated for their cytotoxicity against four tumor cell lines.

## 1. Introduction

Gliotoxin (GT), which represents the most important epidithiodioxopiperazine (ETP) type fungal toxin, is considered a lead agent for anticancer drugs due to its potent cytotoxicity [1,2]. Since the first description by Weindling and Emerson as a metabolite from *Trichoderma lignorum* [3], gliotoxin and its derivatives have been discovered from various fungal species, such as bi(dethio)-10a-methylthio-3a-deoxy-3,3a-didehydrogliotoxin and 6-deoxy-5a,6-didehydrogliotoxin from *Aspergillus fumigatus* [2], bisdethiobis (methylthio) gliotoxin from *Gliocladium deliquescens* [4], and the dimeric eutypellizines N–S from *Eutypella* sp [5]. Furthermore, GT derivatives are well known for their diverse bioactivities, especially the cytotoxicity. Researchers who have focused on the anticancer mechanism of GT derivatives on different cancer cell lines indicated that the intramolecular disulfide bridge makes the most influential contribution to their activities [6]. Nevertheless, to date, to the best of our knowledge, there is no relevant report about gliotoxin dimers which connect through inter-monomer disulfide bridge.

Deep-sea fungi, inhabiting the sediment or water at a depth over 1000 m below the surface, exhibit the potential to metabolize bioactive natural products with fantastic skeletons [7]. Among them, over 50% show cytotoxicity against different human cancer cell lines [7], for example, the cladosins I from *Cladosporium sphaerospermum* can suppress HL-60 cell line with the IC_50_ value of 2.8 μM [8]. Our previous studies suggested that a strain of *Dichotomomyces cejpii* FS110, isolated from deep-sea sediments from South China Sea, tended to produce novel cytotoxic gliotoxin derivatives while being cultured in PDB medium [9]. In this study, further chemical and biological investigations of the extract from solid culture medium of this strain led to the discovery of three dimeric gliotoxin derivatives, dechdigliotoxins A–C (**1–3**), which represented the first examples of gliotoxin dimers constructing an unprecedented exocyclic disulfide linkage, as well as four previously obtained monomers (**4–7**). Herein, the details of the isolation, structure identification, and biological assay of the isolated compounds are discussed.

## 2. Results and Discussion

**Figure 1:**
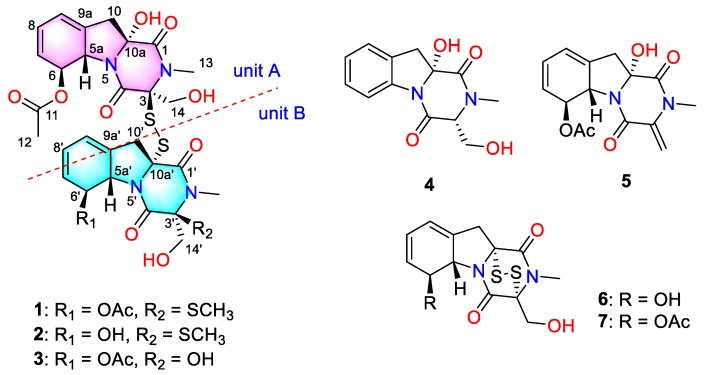
The structures of **1–7**.

The solid fermentation product of the deep-sea-derived fungus *Dichotomomyces cejpii* FS110 was extracted with methanol and then concentrated under reduced pressure to give an extract, which was further subjected to repeated column chromatography to afford compounds **1–7** (Figure 1). The known compounds were identified as dichotocejpins C and B (**4** and **5**, respectively), as well as gliotoxin and acetylgliotoxin (**6** and **7**, respectively) by comparing their spectroscopic data with those previously isolated [9].

Dechdigliotoxin A (**1**) was obtained as a colorless powder with molecular formula of C_31_H_36_N_4_O_11_S_3_ established on the basis of the sodiated HRESIMS peak at m/z 759.1454 [M + Na]^+^ (calcd 759.1440, C_31_H_36_N_4_O_11_S_3_Na). The ^1^H NMR spectrum recorded in DMSO (Table S1) gave the presence of a quaternary hydroxy groups at *δ*_H_ 6.97 (10a-OH, s) and two primary alcohol hydroxy groups at *δ*_H_ 5.28 (14-OH, t, *J* = 5.8) and 5.40 (14′-OH, dd, *J* = 6.5, 5.4); five methyls including two N-methyls at *δ*_H_ 3.03 and 2.96 (13/13′-CH_3_, s); and two hydroxymethyl groups at *δ*_H_ 4.33/3.85 (14-CH_2_, dd) and 4.05/3.61 (14′-CH_2_, dd). ^13^C NMR, as well as HSQC spectra, revealed 31 signals, which could be divided into two sets, each containing five methines, two methyls, two methylene groups, and six quaternary carbons (including three carbonyl carbons). Comprehensive analysis of the 1D NMR and HRESIMS data suggested that dechdigliotoxin A was an unsymmetic dimeric derivative. The characteristic absorption band at 566 cm^−1^ in IR spectrum indicated the existence of a disulfide bond structure feature.

**Figure 2:**
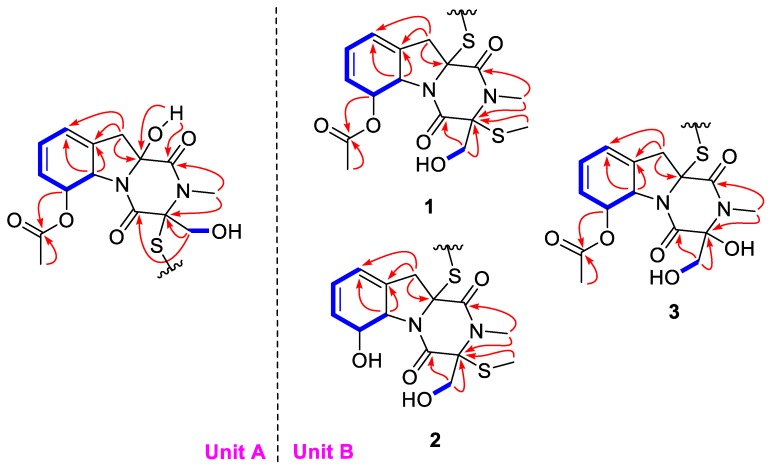
Key HMBC (red arrows) and ^1^H, ^1^H-COSY (blue bold bond) correlation of **1–3**.

The complete structure was further elucidated by the COSY and HMBC correlations (Figure 2). In unit A, the two independent coupling fragments were deduced by the ^1^H, ^1^H-COSY correlations of H-5a/H-6/H-7/H-8/H-9 and H_2_-14/14-OH. The HMBC cross-peaks from 10a-OH to C-1, H_3_-13 to C-1/C-3, as well as H_2_-14 to C-3/C-4, indicated the presence of diketopiperazine moiety. Furthermore, the correlations from H-5a to C-9/C-9a and H_2_-10 to C-5a/C-9/C-9a in HMBC spectrum constructed a gliotoxin derived skeleton of **1**. The acetyl was linked to C-6 based on the detected correlation between H-6 to C-11. In unit B, the COSY and HMBC data indicated the same skeleton as that in unit A. The main difference was that an additional methyl was connected to C-3′ via a sulfur atom, which was evidenced by the chemical shifts and the only HMBC correlation from S-methyl to C-3′. Hence, the planar structure was deduced as shown.

**Figure 3:**
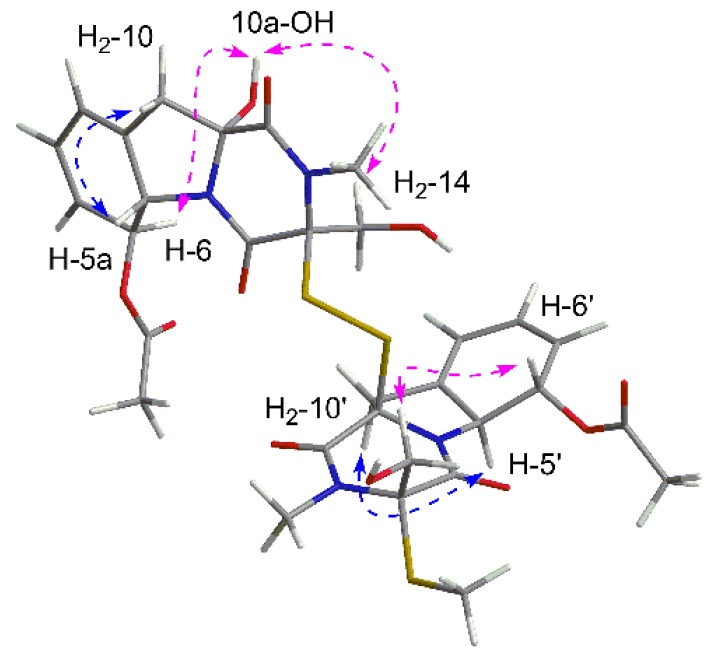
Key NOESY correlations of **1**.

In order to elucidate the relative configuration of **1**, the NOESY spectrum (Figure 3) was recorded in DMSO. In unit A, the lack of correlations between H_2_-10 and H_2_-14 combined with the cross peaks of 10a-OH/H_2_-14 suggested the same side of 10a-OH and the hydroxymethyl, which were assigned to be *α*-oriatation. The NOE effect between H_2_-10 and H-5a was detected, indicating the *β*-oriatation of H-5a. Finally, the large coupling constant (*J* = 14.3 Hz) and the correlation of H-6/10a-OH confirmed the trans configuration of H-5a and H-6. In unit B, the chirality of C-5a′, C-6′, C-10a′ could be easily deduced through NOE correlations and coupling constant, which were the same as those in unit A. Additionally, the correlations between H_2_-14′ and H-6′ revealed the co-facial of them.

**Figure 4:**
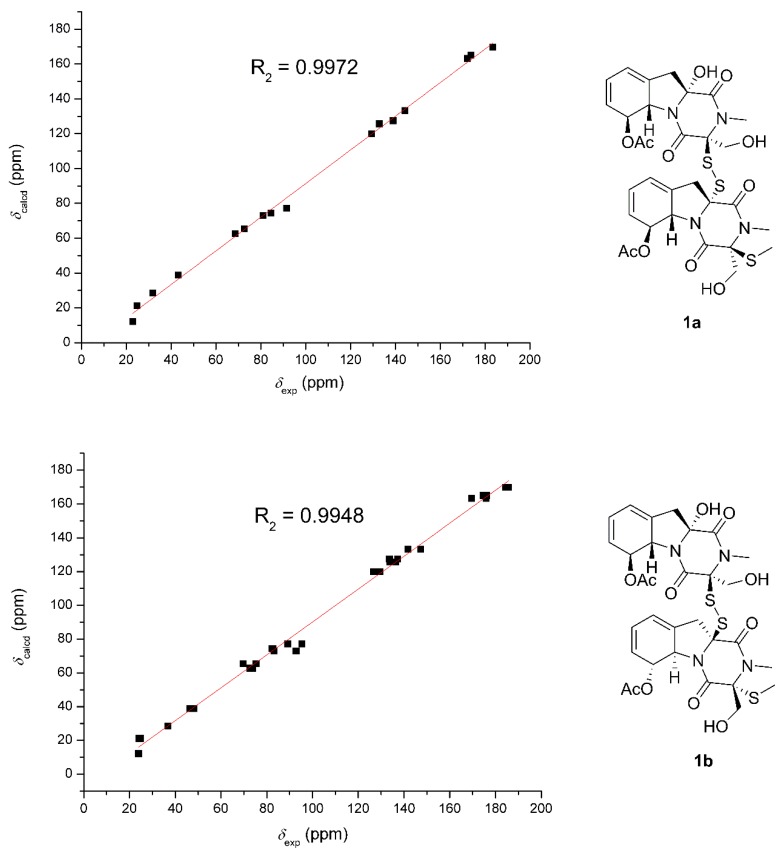
Correlation plots of experimental and calculated ^13^C NMR, as well as DP4+ simulation of **1a** and **1b**.

**Figure 5:**
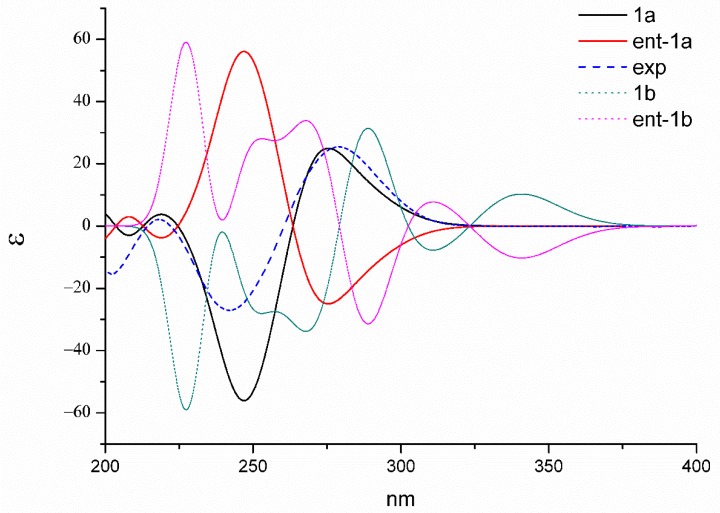
Experimental and calculated ECD spectra of **1a**/ent-**1a** and **1b**/ent-**1b**.

Because of the lack of convincible NOE correlations, the relative configuration between the two units could not be determined exactly. Hence, the theoretical ^13^C NMR chemical shifts calculations and the DP4+ probability simulation of two possible relative structures (**1a** and **1b**) were carried out. Conformers within the 5 kcal mol^−1^ energy window were generated to the b3lyp/6-31+g(d,p) level. Optimized conformers with the Boltzmann distribution over 2% were chosen for NMR chemical shifts calculations in dimethyl sulphoxide at the mPW1PW91/6-311+g(d,p) level. The result (Figure 4) indicated that **1a** exhibited a better correlation coefficient (R^2^: 0.9972) and mean absolute errors (MAE) value (9.5 ppm) as compared with those of **1b** (R^2^: 0.9948; MAE = 10.0 ppm). Moreover, DP4+ simulation suggested **1a** should be the true structure with 99.89% probability. Then, the absolute configuration of **1** was elucidated by performing a quantum chemical calculation of the ECD spectra at the b3lyp/6-311+g(d,p) level. The optimized conformers of both **1a** and **1b** were selected based on the previous NMR calculations. The results shown in Figure 5 suggested that the calculated plot of **1a** exhibited a better fit to the experimental spectrum. Thus, the absolute configuration was assigned as 3*S*, 5a*S*, 6*S*, 10a*R*, 3′*S*, 5a′*S*, 6′*S,* and 10a′*R*.

Dechdigliotoxin B (**2**), which was obtained as a colorless powder, gave the molecular formula of C_29_H_34_N_4_O_10_S_3_ based on the sodiated HRESIMS peak at m/z 717.1323 [M + Na]^+^ (calcd 717.1335, C_29_H_34_N_4_O_10_S_3_Na). The ^1^H and ^13^C NMR spectra recorded in methanol were nearly identical to those of **1** and the main difference was that the methyl signal *δ*_H_ 2.00 (12′-Me) and the carbonyl carbon *δ*_C_ 169.8 (C-11′) were absent. Moreover, the obvious upfield shifts of H-5a′ and H-6′ (from *δ*_H-5a′_ 5.18 and *δ*_H-6′_ 6.14 in **1** to *δ*_H-5a′_ 4.96 and *δ*_H-6′_ 4.86 in **2**, respectively) were observed, suggesting that dechdigliotoxin B was a deacetylation product of **1** at C-6′. Further analysis of 2D NMR data confirmed the planar structure as shown (Figure 2).

**Figure 6:**
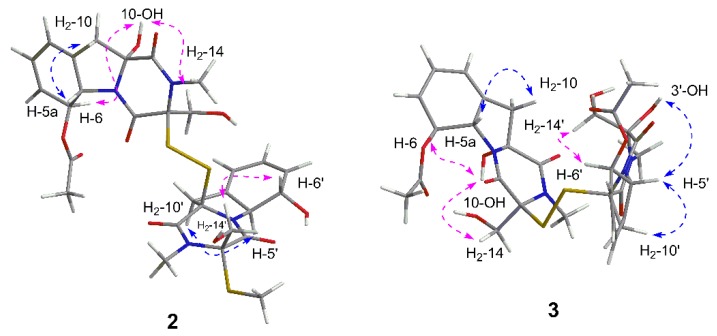
Key NOESY correlations of **2** and **3** recorded in DMSO.

**Figure 7:**
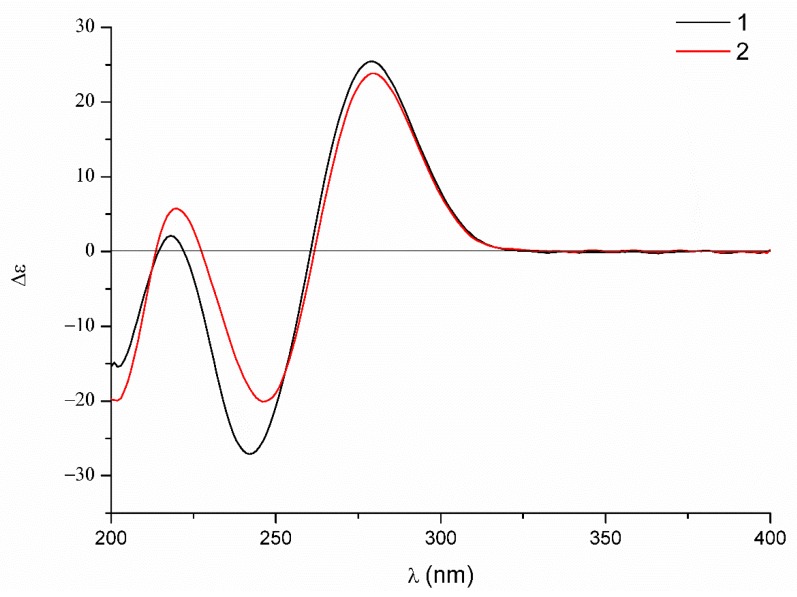
Experimental ECD spectra of **1** and **2**.

The relative configuration was elucidated based on the NOESY correlations recorded in dimethyl sulfoxide-*d*_6_ (Figure 6) and the coupling constants, which were the same as those of dechdigliotoxin A. Additionally, the identical cotton effects in the experimental ECD spectra (Figure 7) shared by **1** and **2** indicated the same absolute configuration of them. Hence, the complete structure of dechdigliotoxin B was deduced.

Dechdigliotoxin C (**3**) was isolated as a colorless powder, of which the molecular formula was deduced as C_30_H_34_N_4_O_12_S_2_ on the basis of the sodiated ion peak at m/z 729.1519 [M + Na]^+^ (calcd 717.1615, C_30_H_34_N_4_O_12_S_2_Na) in HRESIMS. The similar 1D NMR data (Table 1) and the characteristic IR absorption band at 555 cm^−1^ as compared to those of **1** suggested that they shared the same framework except the substituent at C-3′. The absence of S-methyl (*δ*_H_ 2.11; *δ*_C_ 13.0) and the downfield shift of C-3′ (*δ*_C_ 88.2) indicated that the SMe in unit B was replaced by a hydroxy group. The further HMBC correlations (Figure 2) from H_3_-13′ to C-1′/C-3′ and from H_2_-14′ to C-3′/C-4′ confirmed the planar structure of dechdigliotoxin C. The relative configurations of unit A and B were elucidated to be the same as those of **1** and **2** on the basis of NOESY correlation measured in dimethyl sulfoxide-*d_6_* (Figure 6).

**Figure 8:**
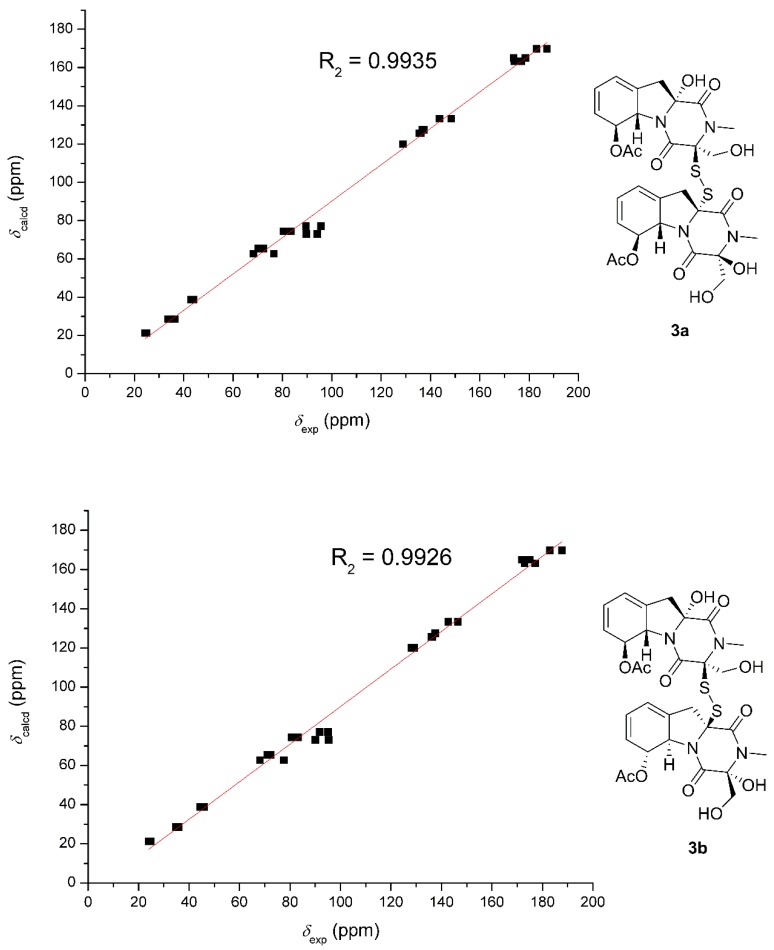
Correlation plots of experimental and calculated ^13^C NMR, as well as DP4+ simulation of **3a** and **3b**.

**Figure 9:**
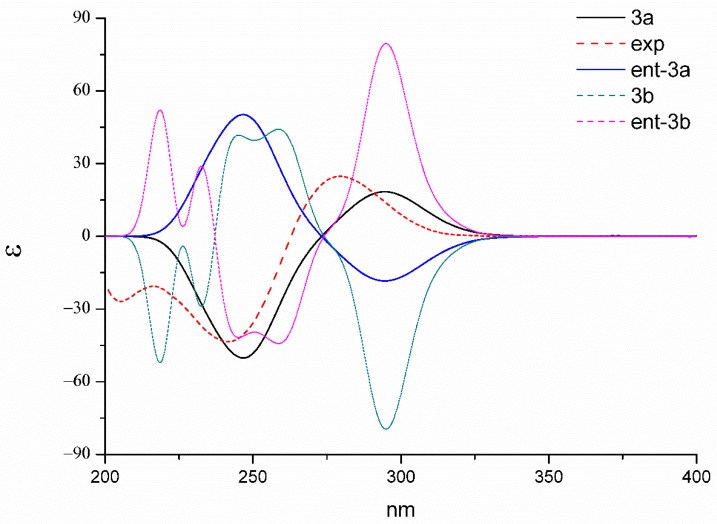
Experimental and calculated ECD spectra of **3a**/ent-**3a** and **3b**/ent-**3b**.

The relative structure between the two monomers were determined based on the ^13^C NMR chemical shift calculations and DP4+ simulation (Figure 8). The method used was the same as those adopted in **1**. The R^2^ of **3a** was 0.9935, which was slightly better than that of **3b** (0.9926). DP4+ (chemical shift of carbon data) suggested **3a** should be the likely candidate with 99.5% probability. Finally, the identical experimental ECD spectra suggested the same absolute configuration of **3**. In order to further confirm the stereochemistry of dechdigliotoxin C, the theoretical ECD spectra of the two enantiomers (**3a** and ent-**3a**) were also calculated at the b3lyp/6-311+g(d,p) level (Figure 9). The experimental spectrum exhibited an excellent fit to the theoretical plot of **3**, which confirmed the absolute configuration.

**Scheme 1:**
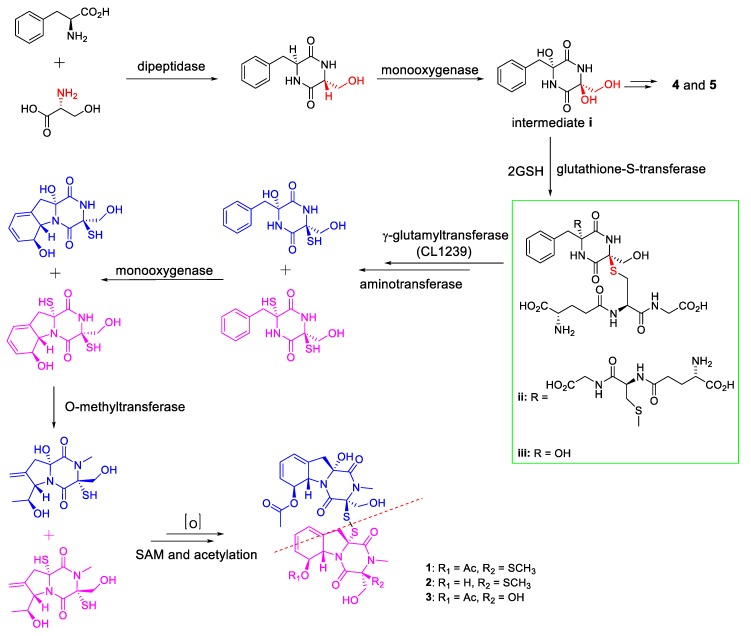
Possible biosynthesis pathway of **1**–**3**.

Cyclic phenylalanylserine cyclo (L-Pshe-L-Ser) is a known biosynthetic precursor of the epidithiodioxopiperazine gliotoxin [10], however, the obtained new metabolites, dechdigliotoxins A–C, in this study, which exhibited a converse chirality at C-3′ as compared with those reported gliotoxin analogues, were suspected to be generated from unusual L-Phe and D-Ser. The possible biosynthesis pathway was shown in Scheme 1. The cyclo-phenylalanyl-serine (L-Phe-D-Ser) was constructed through the dipeptidase. Then, the hydroxylation of *α*-protons of the two amino acid were performed with retention of the chirality of C-3′ and C-10a′ (intermediate i), which would metabolize **4** and **5**. The glutathione-S-transferase should be the key enzyme contributed to the sulfuriztion at C-3′ or/and C-10a′, giving intermediates **ii** and **iii**. Followed by the monooxygenase and O-methyltransferase, the two precursor units A and B were formed from intermediates **i** and **ii**. Finally, the formation of disulfide bond between the two units which constructed **1**–**3** was catalyzed by oxidoreductase. Since the isolated dimers (**1**–**3**) and the monomer (**5**, of which the absolute configuration was confirmed by X-ray diffraction, no. CCDC 1491672, Figure S28) shared the same precursor of intermediate **i**, the stereochemistry could be further evidenced by the same biogenetic consistency.

As it is known that gliotoxin exhibits strong cytotoxicity against cancer cell lines, as well as the normal cell lines, hence, the dimeric analogues **1**–**7** were evaluated for their cytotoxic effects against SF-268, MCF-7, HepG-2, and NCI-H460 cancer cell lines using cisplatin as a positive control. However, none of them showed effects against the tested cell lines except for the gliotoxin (**6**) and acetyl-gliotoxin (**7**) (Table 1). According to the primary structure-activity relationship, we speculated that when the intramolecular disulfide bridge was deficient or converted to an “intermolecular-like” in the dimer, the cytotoxicity would reduce significantly. This would offer a new idea to reduce the toxicity of gliotoxin derivatives toward the normal cell lines. On the other hand, more bioactivities, not limited to the cytotoxicity, would be screened for the new dimers **1**–**3** in the future.

## 3. Materials and Methods

### 3.1. General

HRESIMS were measured on a Thermo MAT95XP high resolution mass spectrometer (Thermo Fisher Scientific, Bremen, Germany). Optical rotations were recorded using an Anton Paar MCP-500 (Anton Paar, Graz, Austria) and the electronic circular dichroism (ECD) measured on a Jasco 820 spectropolarimeter (Jasco Corporation, Kyoto, Japan) under N_2_ gas protection. IR and UV spectra were recorded on Shimadzu IR Affinity-1 and Shimadzu UV-2600 (Shimadzu Corporation, Kyoto, Japan) spectrophotometer, respectively. NMR measurements were carried out on a Bruker Avance-500M spectrometer (Bruker, Fällanden, Switzerland) with tetramethylsilane as an internal standard. Shimadzu LC-20 AT (Shimadzu Corporation, Kyoto, Japan) equipped with an SPD-M20A PDA detector (Shimadzu Corporation, Kyoto, Japan) was adopted for semipreparative HPLC separation with YMC-pack SIL and YMC-pack ODS-A column (250 × 20 mm, 5 μm, 12 nm, YMC CO., Ltd., Kyoto, Japan). Silica gel (200–300 mesh) and Sephadex LH-20 gel were purchased from Qingdao Marine Chemical Plant (Qingdao, China) and Amersham Biosciences (Uppsala, Sweden), respectively. All solvents were of analytical grade (Guangzhou Chemical Regents Company, Ltd., Guangzhou, China).

### 3.2. Material and Identification

The fungal strain *D. cejpii* FS110 was isolated from deep-sea sludge in the South China Sea (19° 0.368′ N, 117° 58.233′ E; depth 3941 m), which was identified as *Dichotomomyces cejpii* according to morphological traits and ITS rDNA sequence analysis. The sequence data have been submitted to GenBank, accession number KF706672. The details of the genetic and deposit information could be found in our previously published reference [9].

### 3.3. Fermentation, Extraction, and Isolation

A grown PDA culture of *D. cejpii* FS110 was used for preparation of the seed cultures, which was further inoculated in 250 mL of PDB (potato 200 g/L, glucose 20 g/L, KH_2_PO_4_ 3 g/L, MgSO_4_•7H_2_O 1.5 g/L, vitamin B_1_ 10 mg/L, sea salt 15 g/L) in a 250 mL Erlenmeyer flask. After incubating for 5 days in a rotary shaker (200 rpm) at 28 °C, the seed culture was transformed to solid medium (30 Erlenmeyer flask each containing 250 g rice and 400 mL saline water). After stationary incubating for 30 days at room temperature, the fermentation product was extracted with methanol to yield a dark brown oily residue (40.0 g), which was further subjected to column chromatography on silica gel eluted with petroleum ether/EtOAc in linear gradient (9:1 to 1:3) to give 26 fractions (Fr.1–Fr.26). Fr.7 was subjected to Sephdex-20 washed by methanol to obtain compound **1** (4.8 mg). Fr.9 was subjected to Sephadex LH-20 (methanol/dichloromethane, 1/1, v/v) to give compound **2**, which was further purified by semi-preparative reversed-phase (RP) HPLC system equipped with a YMC column (methanol/water, 85:15, 2 mL/min) (1.4 mg, t*_R_* = 17 min). Fr. 10 was separated by silica gel (methanol/ dichloromethane, 2/98, v/v) to yield **5** (1.0 mg). Fr.11 was subjected to preparative reversed-phase (RP) HPLC system equipped with a YMC column (methanol/water, 60:40, 8 mL/min), then further purified by semi-preparative reversed-phase (RP) HPLC with the same column (acetonitrile/water, 90:10, 2 mL/min) to get **3** (2.1 mg, t_R_ = 9.7 min) and **4** (2.4 mg, t_R_ = 10.9 min).

#### 3.3.1. Dechdigliotoxin A (1)

Colorless amorphous powder; ${\left[\alpha \right]}_{D}^{25}$ + 99.9 (c 0.01, mathanol); UV (MeOH) λ_max_ (logε): 220 (4.15), 265 (2.11) nm. IR (KBr): 3270, 2944, 2850, 1732, 1721, 1690, 1680, 1675, 1298, 1206, 1011, 566 cm^−1^; HRESIMS m/z 759.1454 [M + Na]^+^ (calcd 759.1440, C_31_H_36_N_4_O_11_S_3_Na); ^1^H and ^13^C NMR data: see Table S1 and Figure S1–S6.

#### 3.3.2. Dechdigliotoxin B (2)

Colorless amorphous powder; ${\left[\alpha \right]}_{D}^{25}$ + 85.4 (c 0.01, mathanol); UV (MeOH) *λ*_max_ (logε): 219 (4.18), 263 (2.10) nm; IR (KBr): 3266, 2951, 2790, 1720, 1698, 1677, 1676, 1241, 1216, 1021, 563 cm^−1^; HRESIMS m/z 717.1323 [M + Na]^+^ (calcd 717.1335, C_29_H_34_N_4_O_10_S_3_Na); ^1^H and ^13^C NMR data: see Table S1 and Figure S7–S13.

#### 3.3.3. Dechdigliotoxin C (3)

Colorless amorphous powder; ${\left[\alpha \right]}_{D}^{25}$ − 109.7 (c 0.01, methanol); UV (MeOH) *λ*_max_ (logε): 221 (3.98),269 (1.97) nm. IR (KBr): 3199, 2898, 2760, 1720, 1709, 1688, 1682, 1679, 1240, 1206, 1031, 555 cm^−1^; HRESIMS m/z 729.1529 [M + Na]^+^ (calcd 717.1615, C_30_H_34_N_4_O_12_S_2_Na); ^1^H and ^13^C NMR data: see Table S1 and Figure S14–S24.

### 3.4. Quantum Chemical Calculation of NMR Chemical Shifts and ECD Spectrra

Merck molecular force field (MMFF) and DFT/TD-DFT calculations were carried out using the Spartan’14 software (Wavefunction Inc., Irvine, CA, USA) and the Gaussian 09 program, respectively [11]. Conformers which had an energy window lower the 5 kcal mol^−1^ were generated and optimized using DFT calculations at the b3lyp/6-31+g(d,p) level. Frequency calculations were performed at the same level to confirm that each optimized conformer was a true minimum and to estimate their relative thermal free energy (ΔG) at 298.15 K. Conformers with the Boltzmann distribution over 2% were chosen for ^13^C NMR and ECD calculations at the mPW1PW91/6-311+g(d,p) and the b3lyp/6-311+g(d,p) level, respectively. Additionally, solvent effects were considered based on the self-consistent reaction field (SCRF) method with the polarizable continuum model (PCM) [12]. Details of the conformers’ information were provided in Supplementary Materials (Tables S2–S5). The ECD spectrum was generated by the SpecDis program [13] using a Gaussian band shape with 0.22 eV exponential half-width from dipole-length dipolar and rotational strengths. The DP4+ probability simulations were conducted using an applet available at http://www-jmg.ch.cam.ac.uk/tools/nmr/DP4/.

### 3.5. Cytotoxicity Assay

The tested cell lines were purchased from the Chinese Academy of Sciences Cell Bank (Shanghai, China). SF-268, MCF-7, and HepG-2 were selected to be the targeted cancer cell lines and the cytotoxicity were tested based on the previously reported SRB method [14]. Cells (180 μL) with a density of 30,000 cells/mL of media were injected into 96-well plates and incubated at 37 °C for 24 h under 5% CO_2_. Then, different concentrations of the tested compounds (20 μL) were added and further incubated for 72 h. After that, cell monolayers were fixed with 50 μL trichloroacetic acid (wt/v: 50%) and stained with 0.4% SRB (dissolved in 1% CH_3_COOH) for 30 min. The monolayers were washed by 1% CH_3_COOH three times to remove the unbound dye. The mixtures were dissolved in 200 µL Tris base solution (10 mM) and recorded the OD at 570 nm using a microplate reader. Cisplatin was used as a positive control possessing potent cytotoxic activity. All data were obtained in triplicate.

## 4. Conclusions

In summary, seven gliotoxin derivatives were obtained from the deep-sea derived fungus *Dichotomomyces cejpii*. To the best of our knowledge, the three novel dimers dechdigliotoxins A–C (**1**–**3**) represented the first examples of dimeric analogues from nature with an unprecedented exocyclic disulfide linkage. The proposed biosynthetic pathway suggested that they should be generated from the unusual precursors L-Phe and D-Ser. Moreover, the bioassays indicated that when the intramolecular disulfide bridge was deficient or converted to a “intermolecular-like” in the dimer, the cytotoxicity would reduce significantly. This discovery makes a contribution to the chemical diversity of gliotoxins and, for further investigation, offers a new idea to reduce the toxicity of gliotoxins.

## Figures and Tables

**Table 1:** In vitro cytotoxicity against tested cancer cell lines of **1**–**7**.

Compounds	IC_50_ (μM)			
	SF-268	MCF-7	NCI-H460	HepG-2
1–5	>100	>100	>100	>100
6	0.24 ± 0.10	0.08 ± 0.00	0.24 ± 0.01	0.21 ± 0.01
7	0.25 ± 0.03	0.22 ± 0.04	0.32 ± 0.02	0.49 ± 0.07
Cisplatin	2.37 ± 0.35	3.09 ± 0.27	2.43 ± 0.15	1.39 ± 0.18

## Supplementary Materials

The following are available online at https://www.mdpi.com/1660-3397/17/11/596/s1, Figure S1: ^1^H NMR spectrum of **1** in DMSO (600 MHz), Figure S2: ^13^C NMR spectrum of **1** in DMSO (150 MHz), Figure S3: ^1^H-^1^H COSY spectrum of **1** in DMSO (600 MHz), Figure S4: HSQC spectrum of **1** in DMSO(600 MHz), Figure S5: HMBC spectrum of **1** in DMSO(600 MHz), Figure S6: NOESY spectrum of **1** in DMSO (600 MHz), Figure S7: ^1^H NMR spectrum of **2** in MeOD (600 MHz), Figure S8: ^13^C NMR spectrum of **2** in MeOD (600 MHz), Figure S9: ^1^H-^1^H COSY spectrum of **2** in MeOD (600 MHz), Figure S10: HSQC spectrum of **2** in MeOD (600 MHz), Figure S11: HMBC spectrum of **2** in MeOD (600 MHz), Figure S12: ^1^H NMR spectrum of **2** in DMSO (600 MHz), Figure S13: NOESY spectrum of **2** in DMSO (600 MHz), Figure S14: ^1^H NMR spectrum of **3** in MeOD (500 MHz), Figure S15: ^13^C NMR spectrum of **3** in MeOD (500MHz), Figure S16: ^1^H-^1^H COSY spectrum of **3** in MeOD (500 MHz), Figure S17: HSQC spectrum of **3** in MeOD (500 MHz), Figure S18: HMBC spectrum of **3** in MeOD (500 MHz), Figure S19: ^1^H NMR spectrum of **3** in DMSO (600 MHz), Figure S20: ^13^CNMR spectrum of **3** in DMSO (150MHz), Figure S21: ^1^H, ^1^H-COSY spectrum of **3** in DMSO (600 MHz), Figure S22: HSQC spectrum of **3** in DMSO (600 MHz), Figure S23: HMBC spectrum of **3** in DMSO (600 MHz), Figure S24: NOESY spectrum of **3** in DMSO (500 MHz), Figure S25: HRESI TOF MS spectrum of **1**, Figure S26: HRESI TOF MS spectrum of **2**, Figure S27: HRESI TOF MS spectrum of **3**, Figure S28: The crystal structure of **4**, Table S1: 1H and 13C NMR data of **1**–**3**, Table S2: Energy analysis for the Conformers of **1a**, Table S3: Energy analysis for the Conformers of **1b**, Table S4: Energy analysis for the Conformers of **3a**, Table S5: Energy analysis for the Conformers of **3b**.
