# Supplementary material for: Dechdigliotoxins A–C, Three Novel Disulfide-Bridged Gliotoxin Dimers from Deep-Sea Sediment Derived Fungus Dichotomomyces cejpii
Source: Mar Drugs. 2019 Oct 23;17(11):596. doi: 10.3390/md17110596 (PMC6891313; doi:10.3390/md17110596)
Supplement: Supplementary file 1 [file marinedrugs-17-00596-s001.pdf]

SUPPORTING INFORMATION

Dechdigliotoxins A–C, three novel disulfide-~~B~~bridged gliotoxin dimers from deep-sea sediment derived fungus *Dichotomomyces cejpii*

Zhaoming Liu [1], Zhen Fan [1], Zhanghua Sun [1], Hongxin Liu [1,*], Weimin Zhang [1,*]

[1] State Key Laboratory of Applied Microbiology Southern China, Guangdong Provincial Key Laboratory of Microbial Culture Collection and Application, Guangdong Open Laboratory of Applied Microbiology, Guangdong Institute of Microbiology, Guangdong Academy of Sciences, 100 Central Xianlie Road, Yuexiu District, Guangzhou 510070, China.

* Correspondence: liuhx@gdim.cn; (H.L.); wmzhang@gdim.cn (W.Z.)

Table of contents:

Figure S1. ^{1}H NMR spectrum of **1** in DMSO (600 MHz).
Figure S2. ^{13}C NMR spectrum of **1** in DMSO (150 MHz).
Figure S3. ^{1}H-^{1}H COSY spectrum of **1** in DMSO (600 MHz).
Figure S4. HSQC spectrum of **1** in DMSO (600 MHz).
Figure S5. HMBC spectrum of **1** in DMSO (600 MHz).
Figure S6. NOESY spectrum of **1** in DMSO (600 MHz).
Figure S7. ^{1}H NMR spectrum of **2** in MeOD (600 MHz).
Figure S8. ^{13}C NMR spectrum of **2** in MeOD (600 MHz).
Figure S9. ^{1}H-^{1}H COSY spectrum of **2** in MeOD (600 MHz).
Figure S10. HSQC spectrum of **2** in MeOD (600 MHz).
Figure S11. HMBC spectrum of **2** in MeOD (600 MHz).
Figure S12. ^{1}H NMR spectrum of **2** in DMSO (600 MHz)
Figure S13. NOESY spectrum of **2** in DMSO (600 MHz).
Figure S14. ^{1}H NMR spectrum of **3** in MeOD (500 MHz).
Figure S15. ^{13}C NMR spectrum of **3** in MeOD (500 MHz).
Figure S16. ^{1}H-^{1}H COSY spectrum of **3** in MeOD (500 MHz).
Figure S17. HSQC spectrum of **3** in MeOD (500 MHz).
Figure S18. HMBC spectrum of **3** in MeOD (500 MHz).
Figure S19. ^{1}H NMR spectrum of **3** in DMSO (600 MHz)
Figure S20. ^{13}C NMR spectrum of **3** in DMSO (150 MHz)
Figure S21. ^{1}H, 1H-COSY spectrum of **3** in DMSO (600 MHz)
Figure S22. HSQC spectrum of **3** in DMSO (600 MHz)
Figure S23. HMBC spectrum of **3** in DMSO (600 MHz)
Figure S24. NOESY spectrum of **3** in DMSO (500 MHz).
Figure S25. HRESI TOF MS spectrum of **1**.
Figure S26. HRESI TOF MS spectrum of **2**.
Figure S27. HRESI TOF MS spectrum of **3**.
Figure S28. The crystal structure of **4**.
Table S1. ^{1}H and ^{13}C NMR data of **1**–**3**.
Table S2. Energy analysis for the Conformers of **1a**.
Table S3. Energy analysis for the Conformers of **1b**.
Table S4. Energy analysis for the Conformers of **3a**.
Table S5. Energy analysis for the Conformers of **3b**.

Figure S1. ^{1}H NMR spectrum of **1** in DMSO.

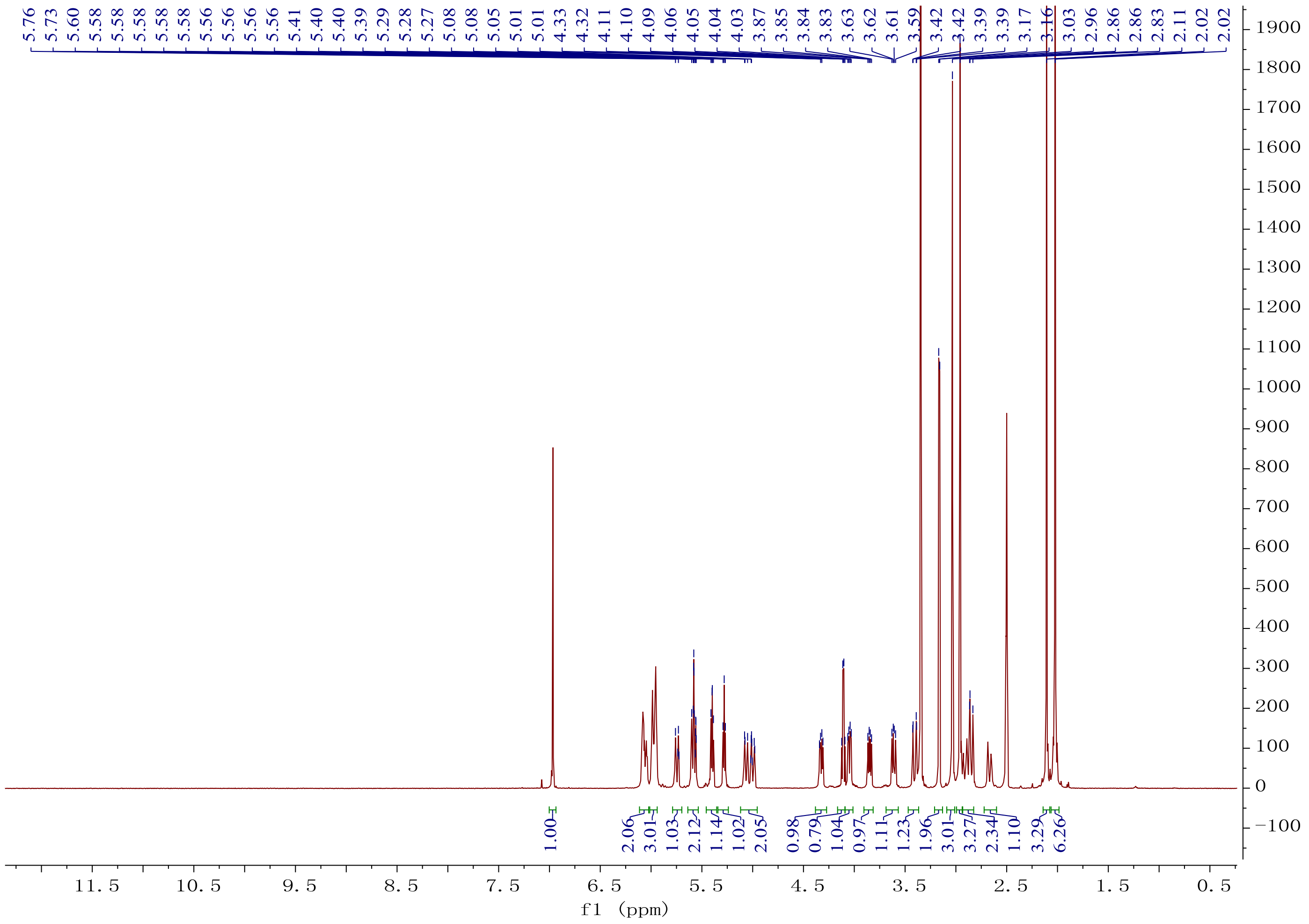

Figure S2. ^{13}C NMR spectrum of **1** in DMSO.

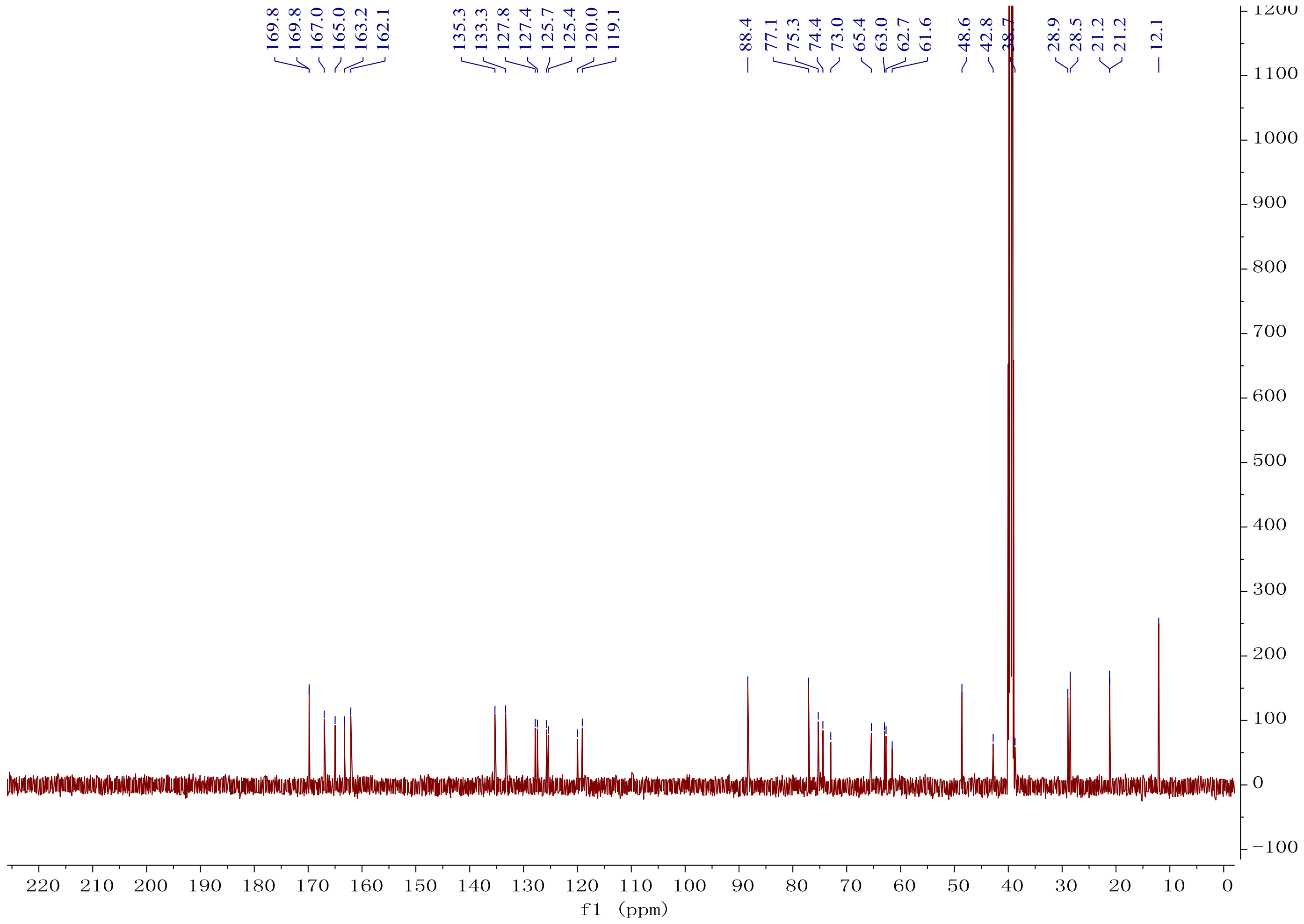

Figure S3. ^{1}H, ^{1}H-COSY spectrum of **1** in DMSO.

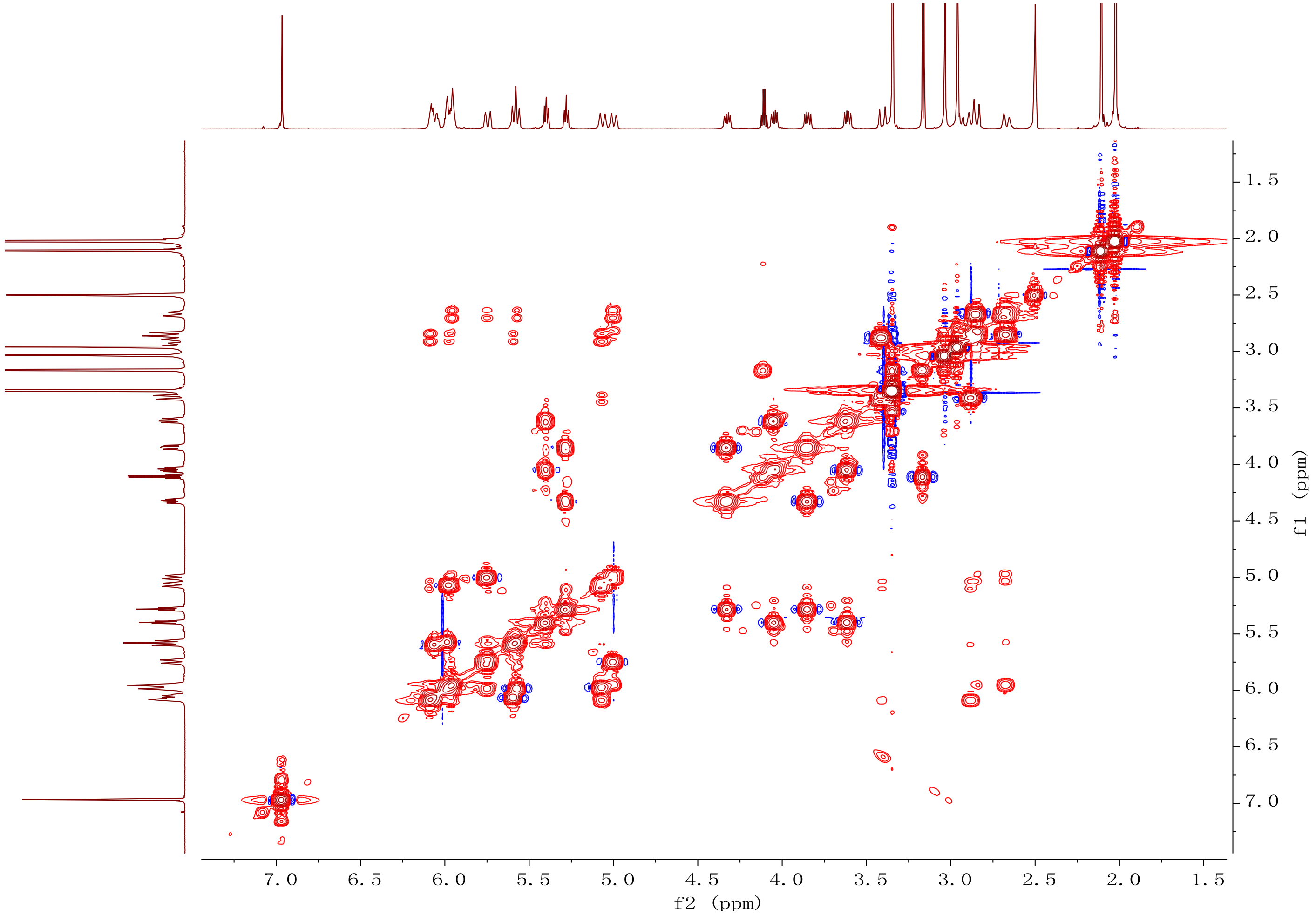

.

Figure S4. HSQC spectrum of **1** in DMSO.

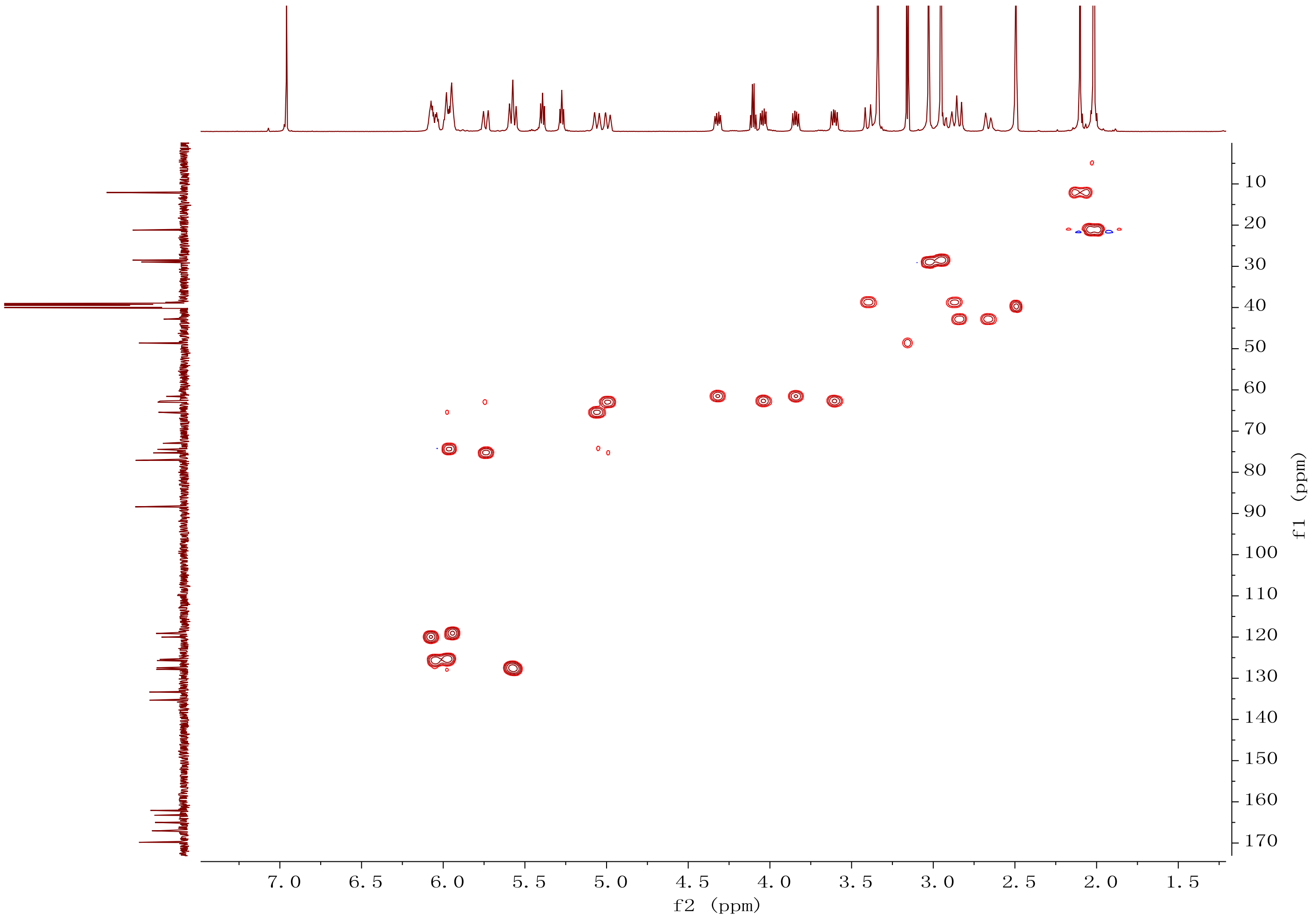

Figure S5. ^{1}H-^{1}H COSY **1** in DMSO.

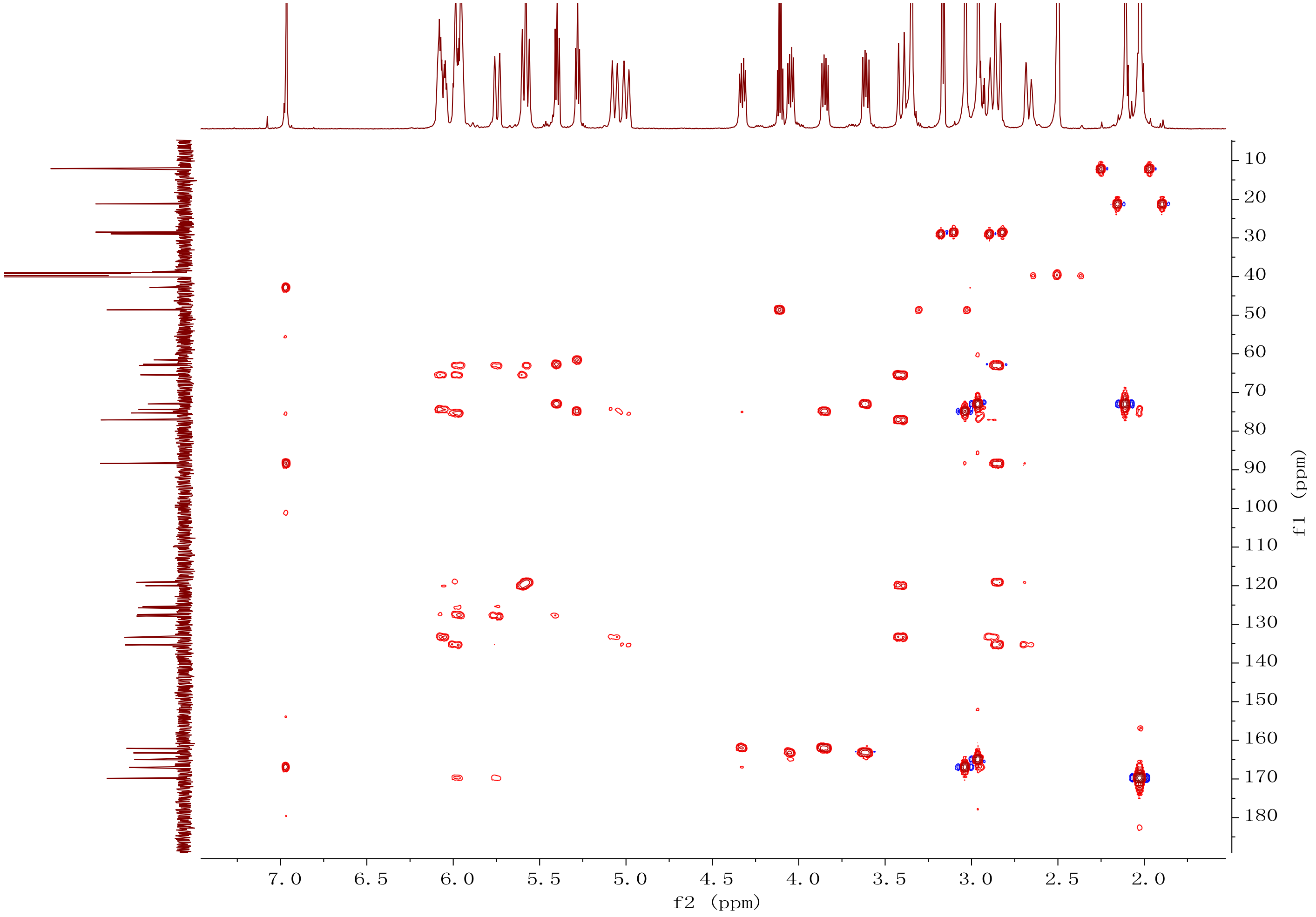

Figure S6. NOESY spectrum of **1** in DMSO.

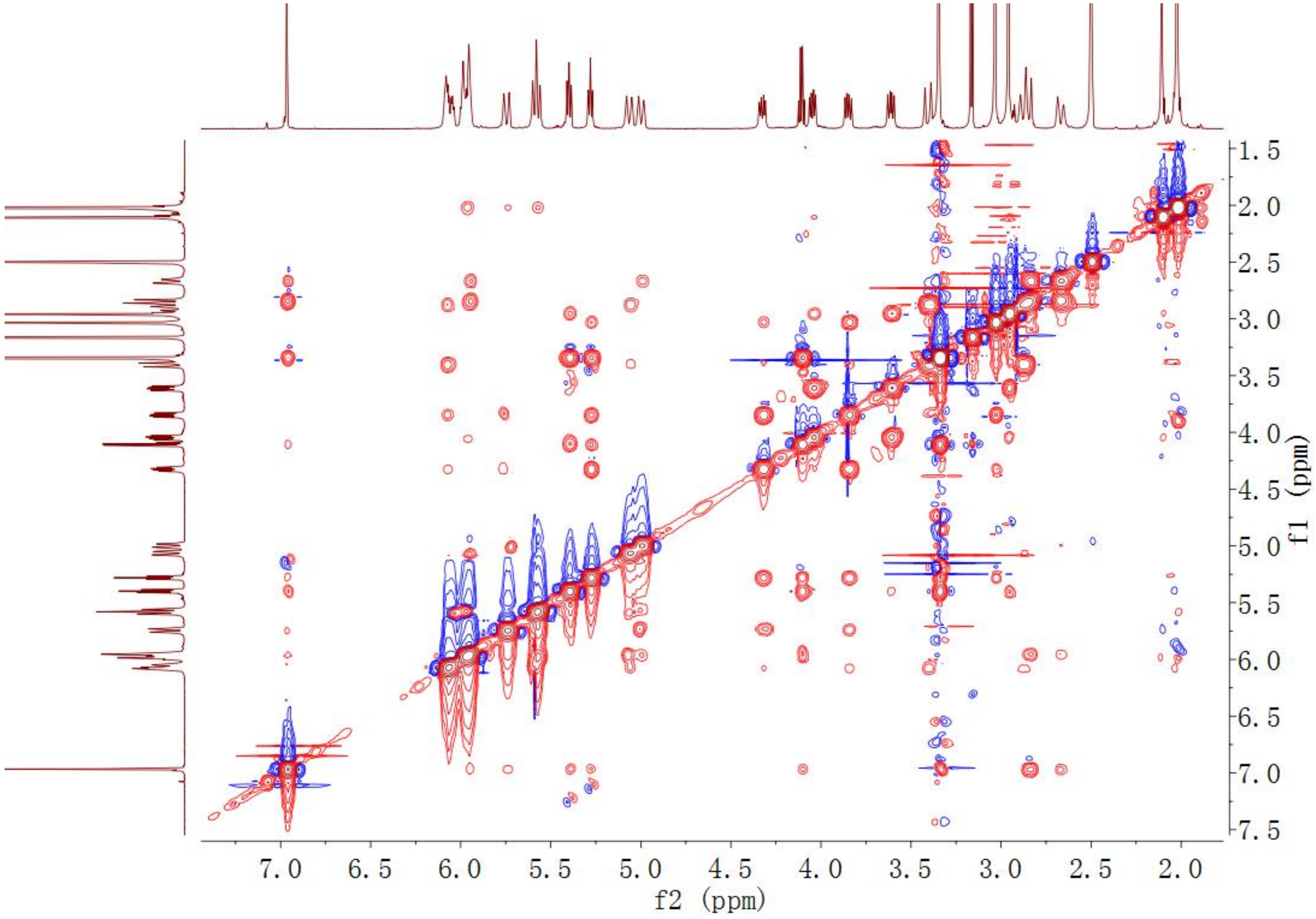

Figure S7. ^{1}H-NMR spectrum of **2** in MeOD.

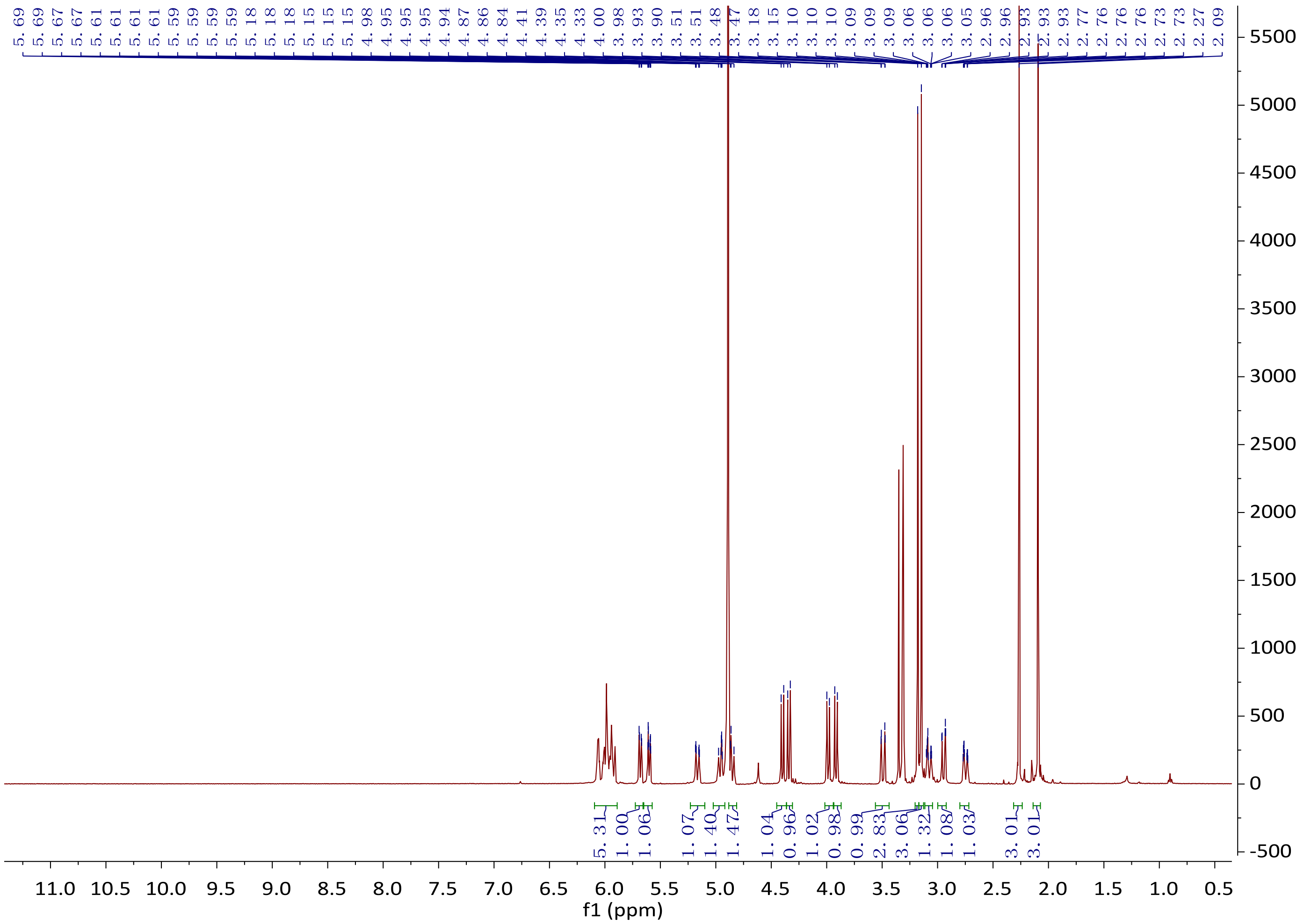

Figure S8. ^{13}C-NMR spectrum of **2** in MeOD.

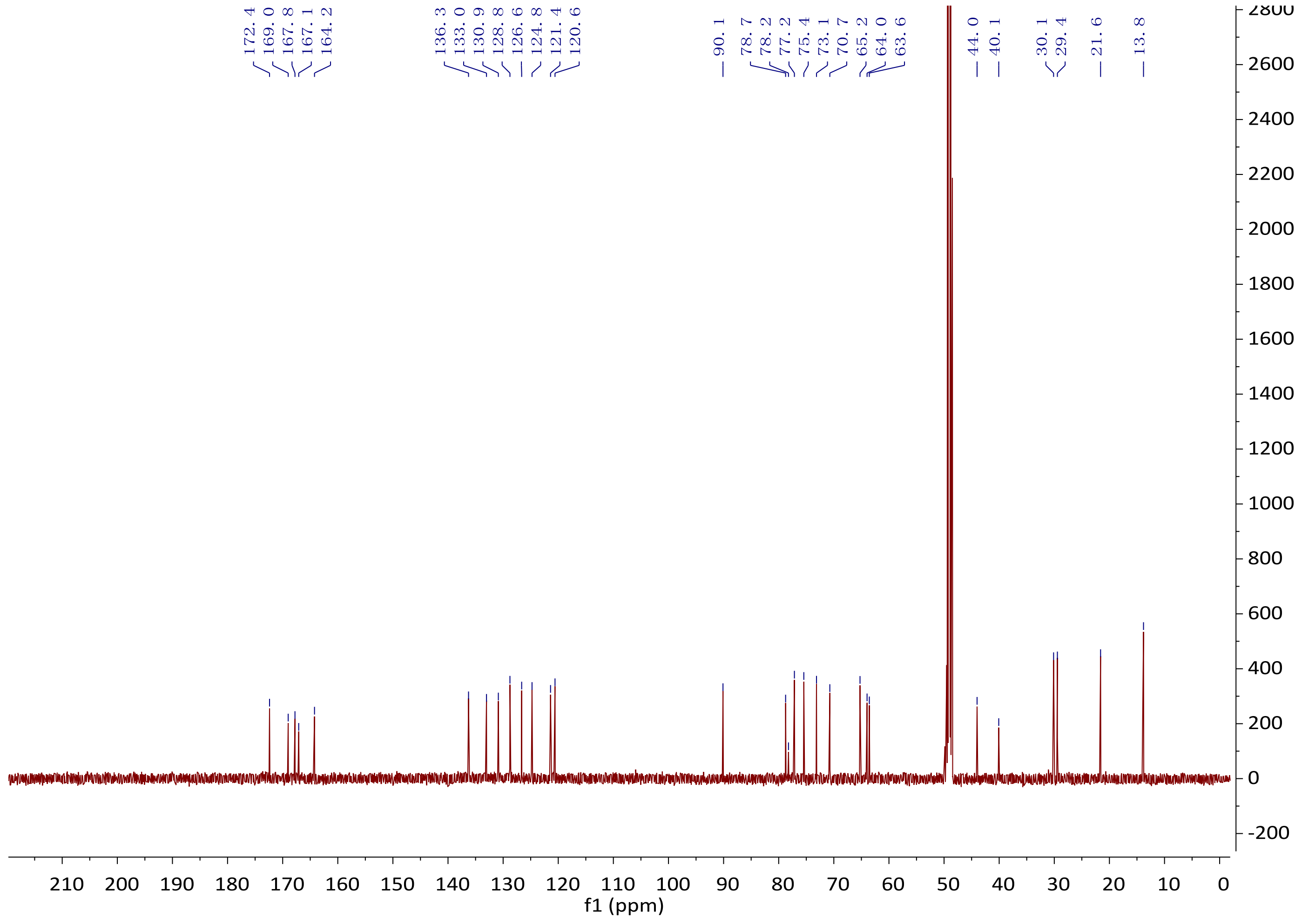

Figure S9. ^{1}H-^{1}H COSY spectrum of **2** in MeOD.

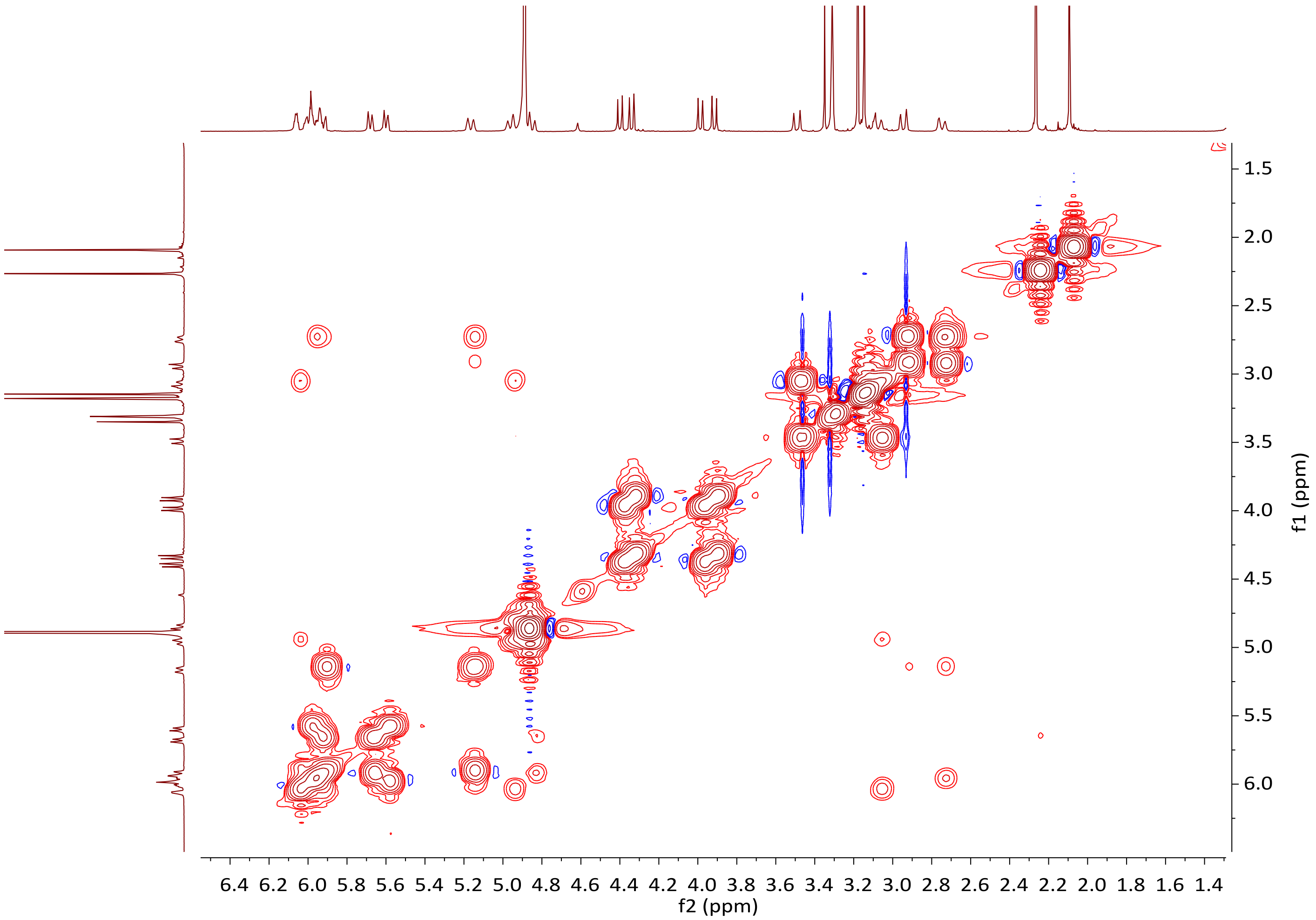

Figure S10. HSQC spectrum of **2** in MeOD.

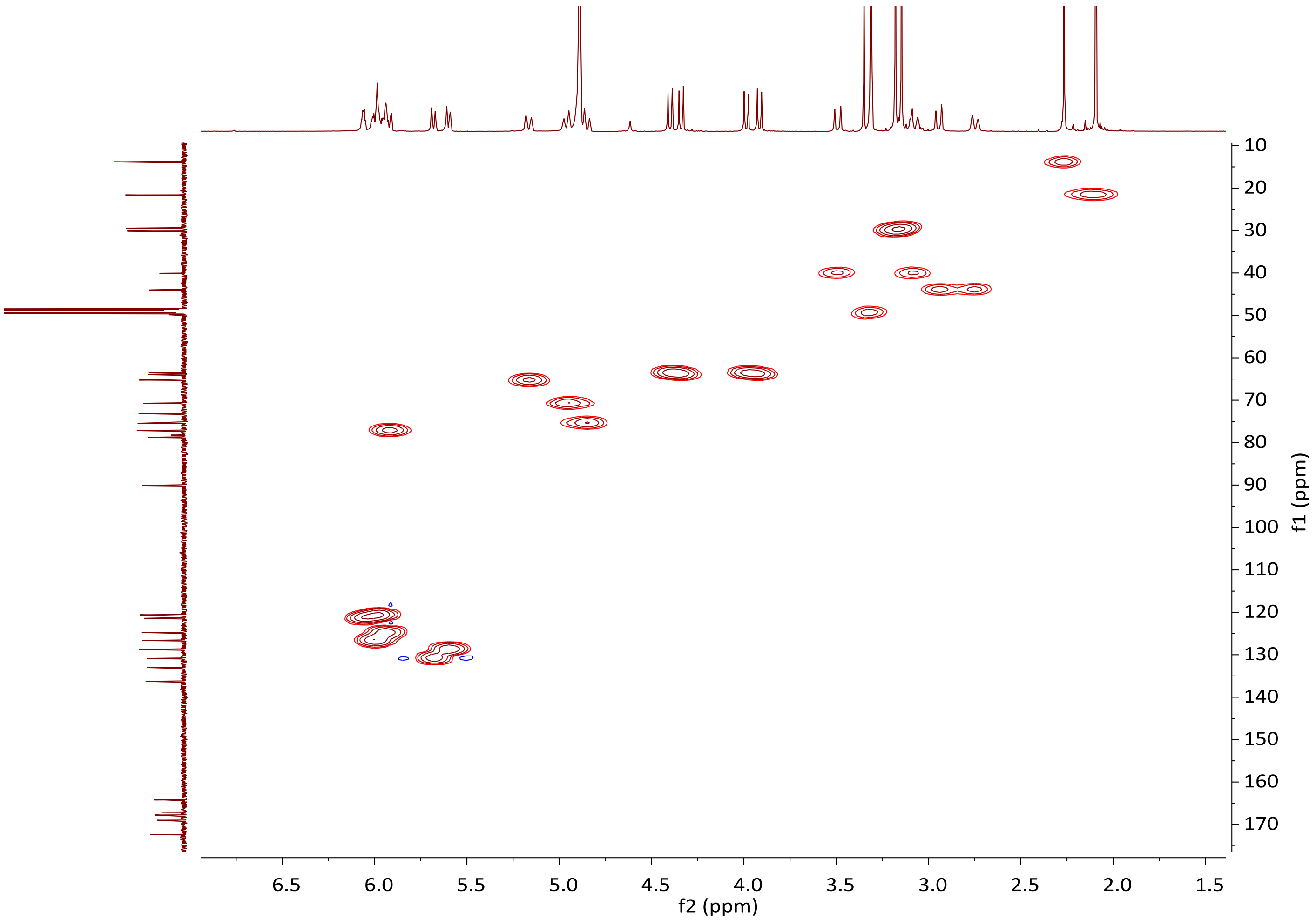

Figure S11. HMBC spectrum of **2** in MeOD.

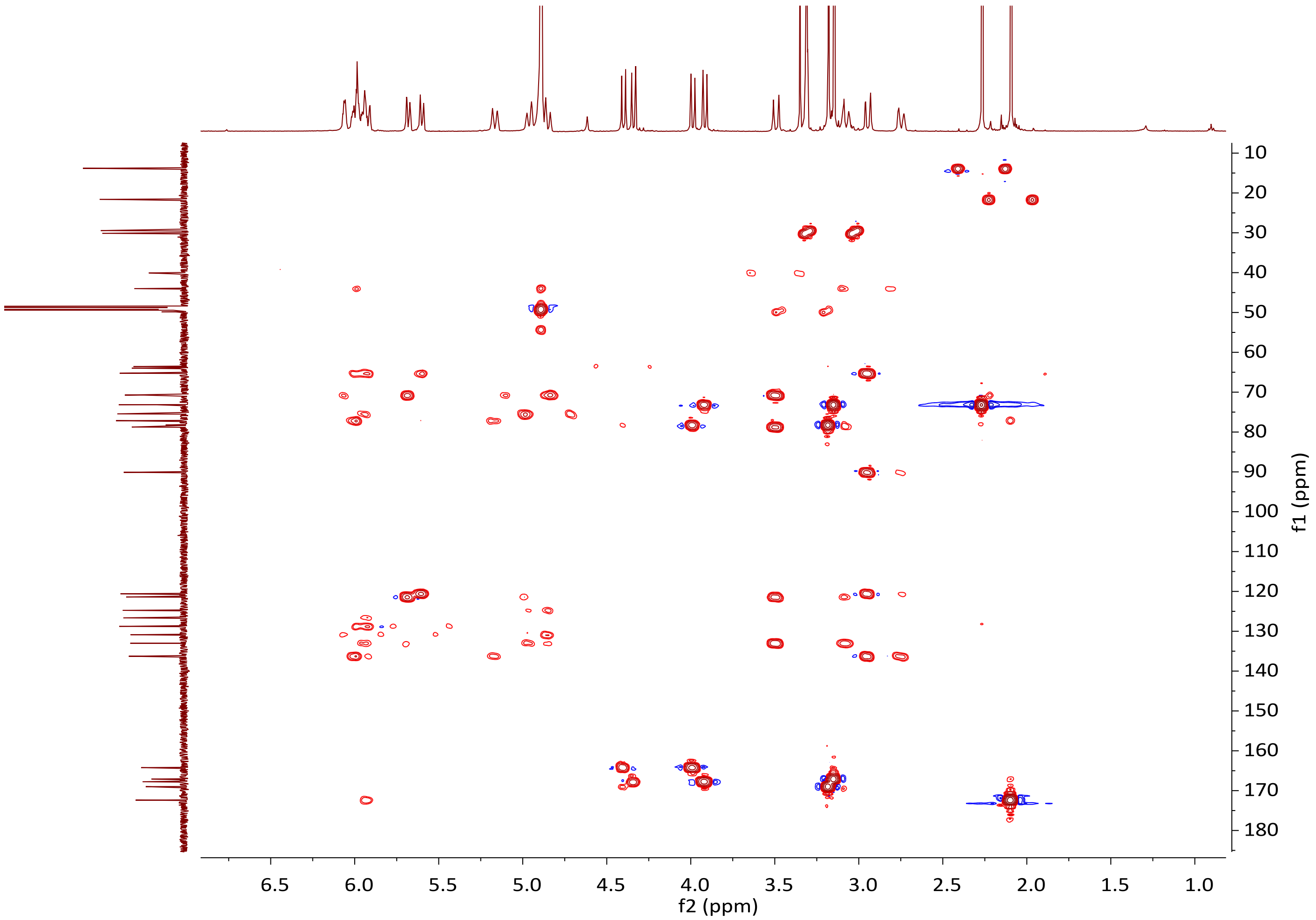

Figure S12. ^{1}H NMR spectrum of **2** in DMSO.

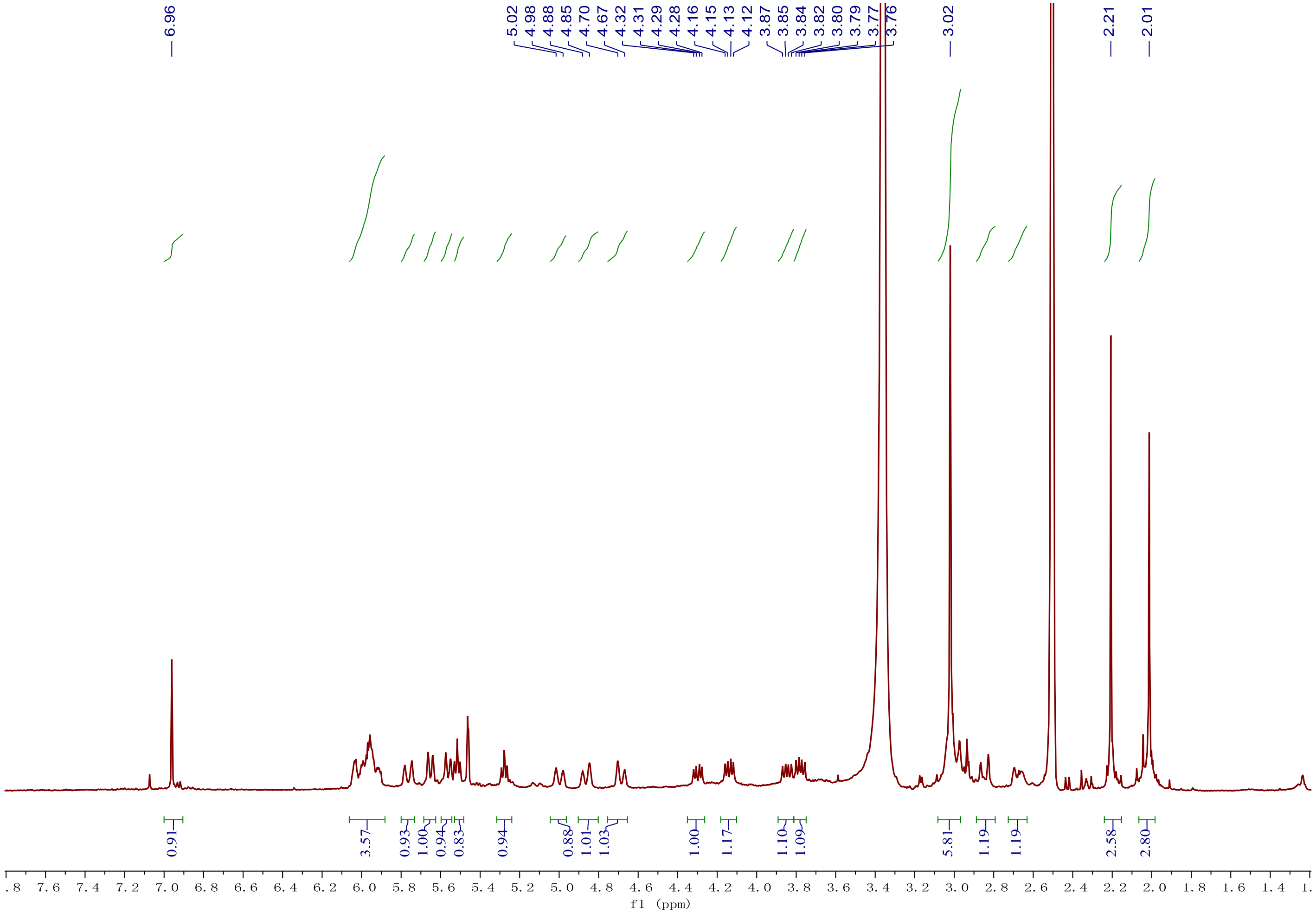

Figure S13. NOESY spectrum of **2** in DMSO.

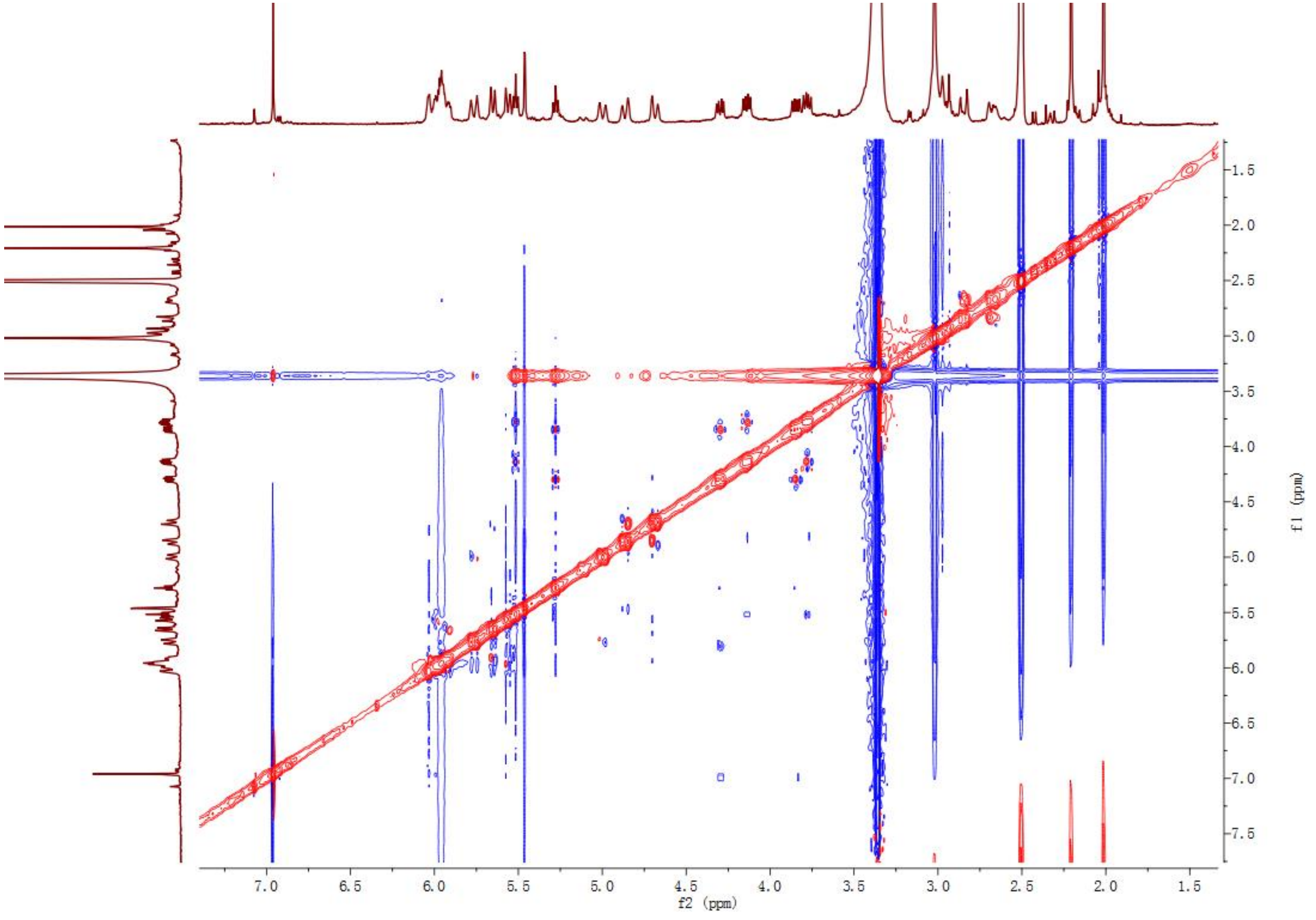

Figure S14. ^{1}H NMR spectrum of **3** in MeOD.

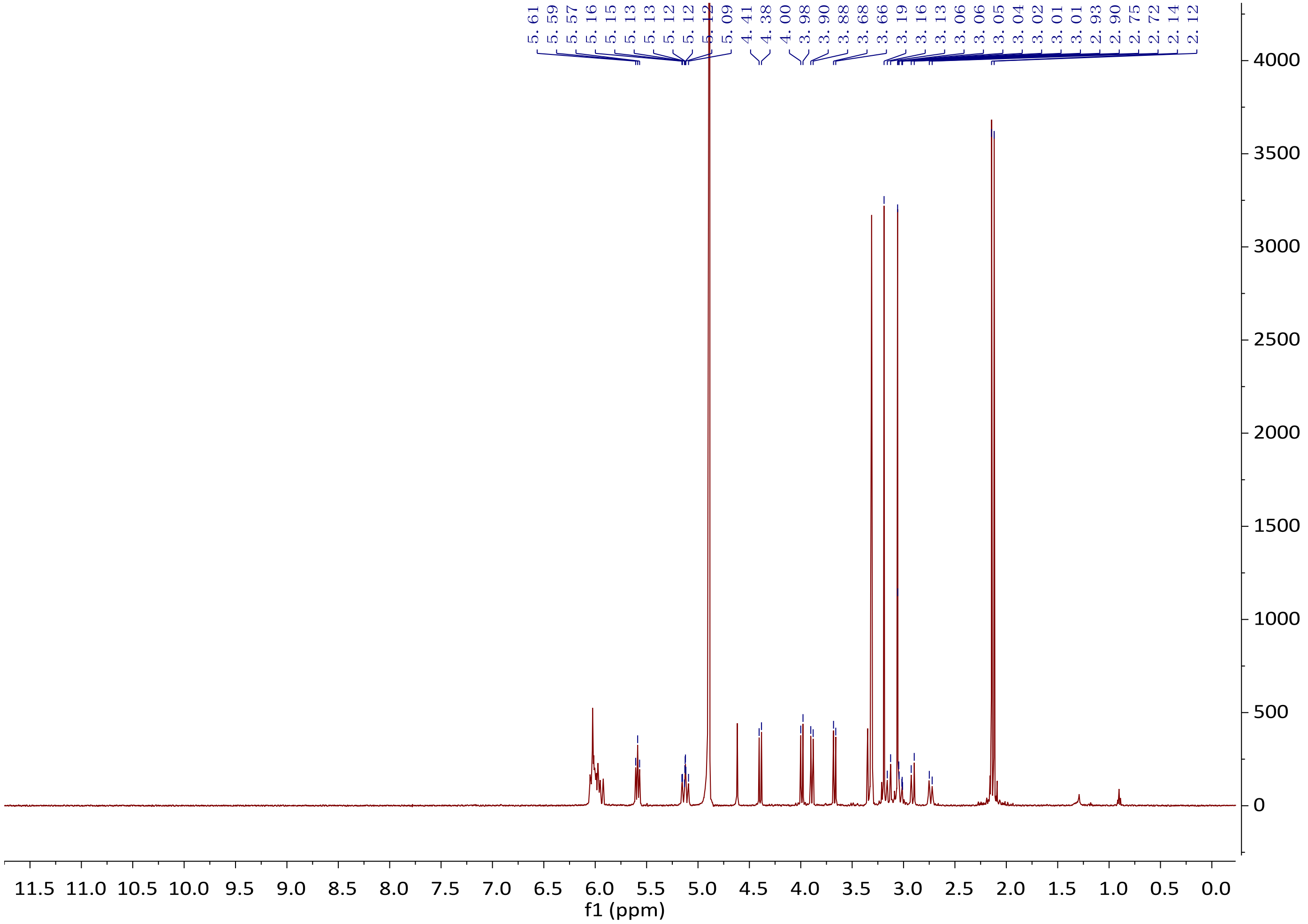

Figure S15. ^{13}C NMR spectrum of **3** in MeOD.

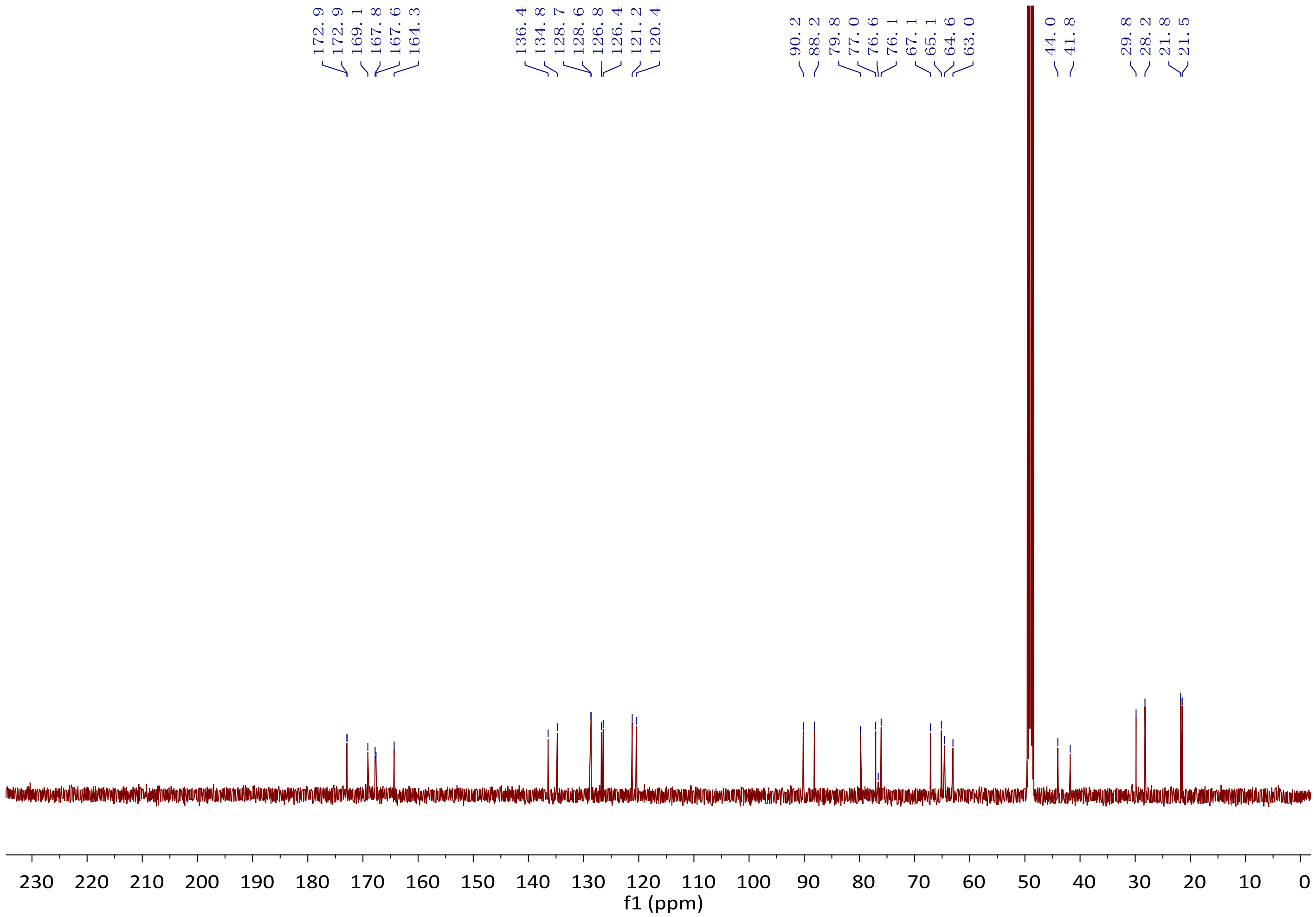

Figure S16. ^{1}H-^{1}H COSY spectrum of **3** in MeOD.

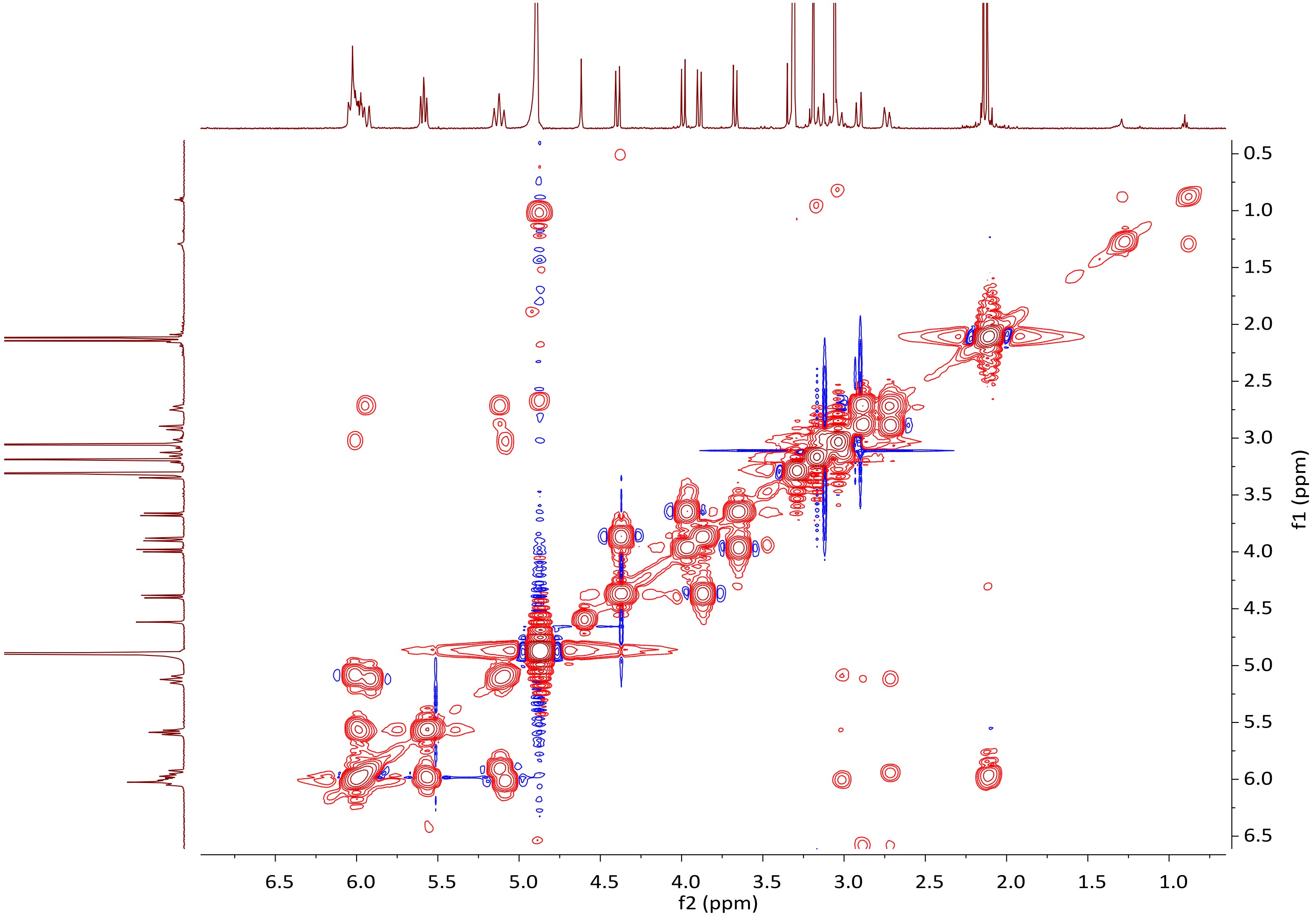

Figure S17. HSQC spectrum of **3** in MeOD.

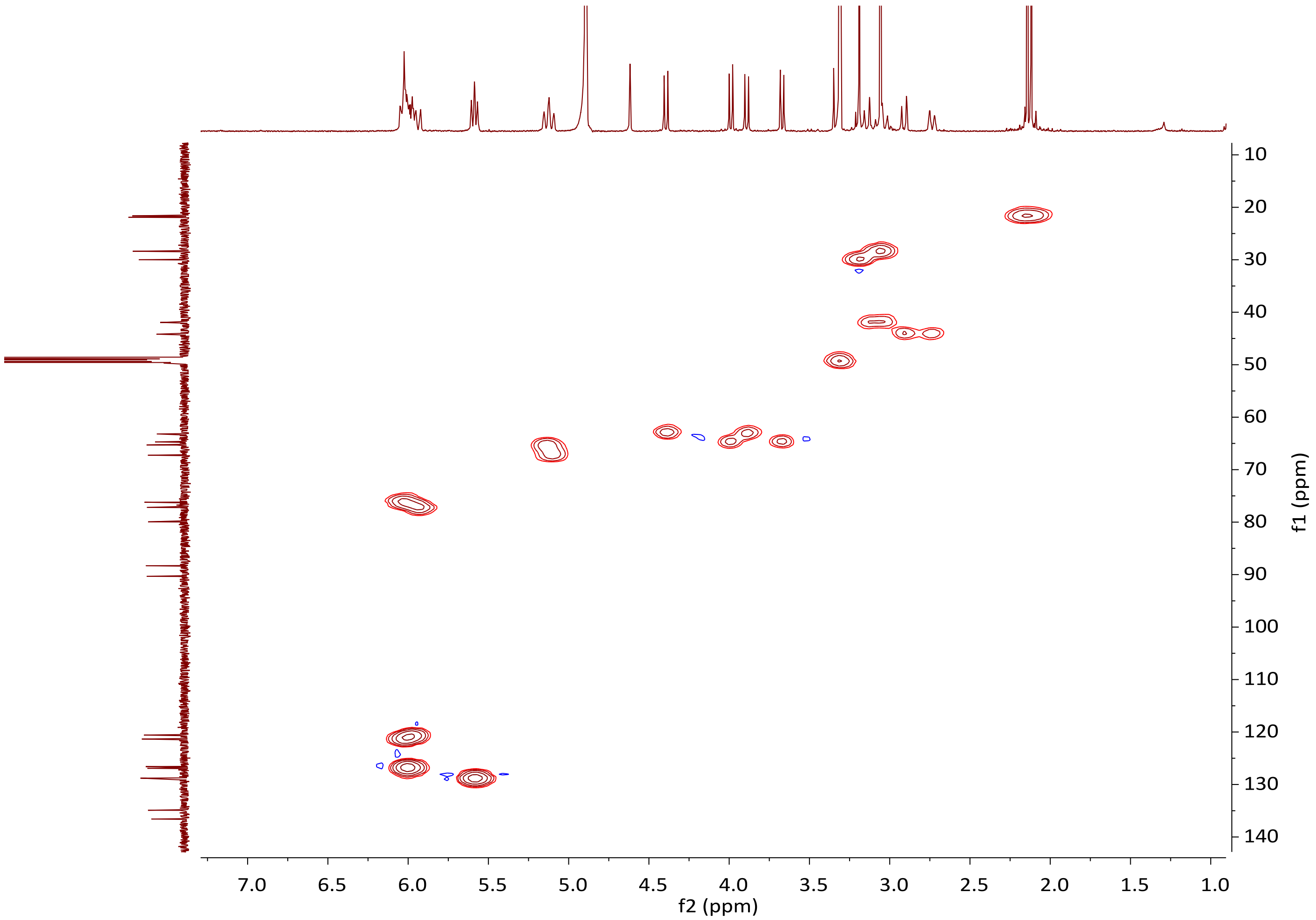

Figure S18. HMBC spectrum of **3** in MeOD.

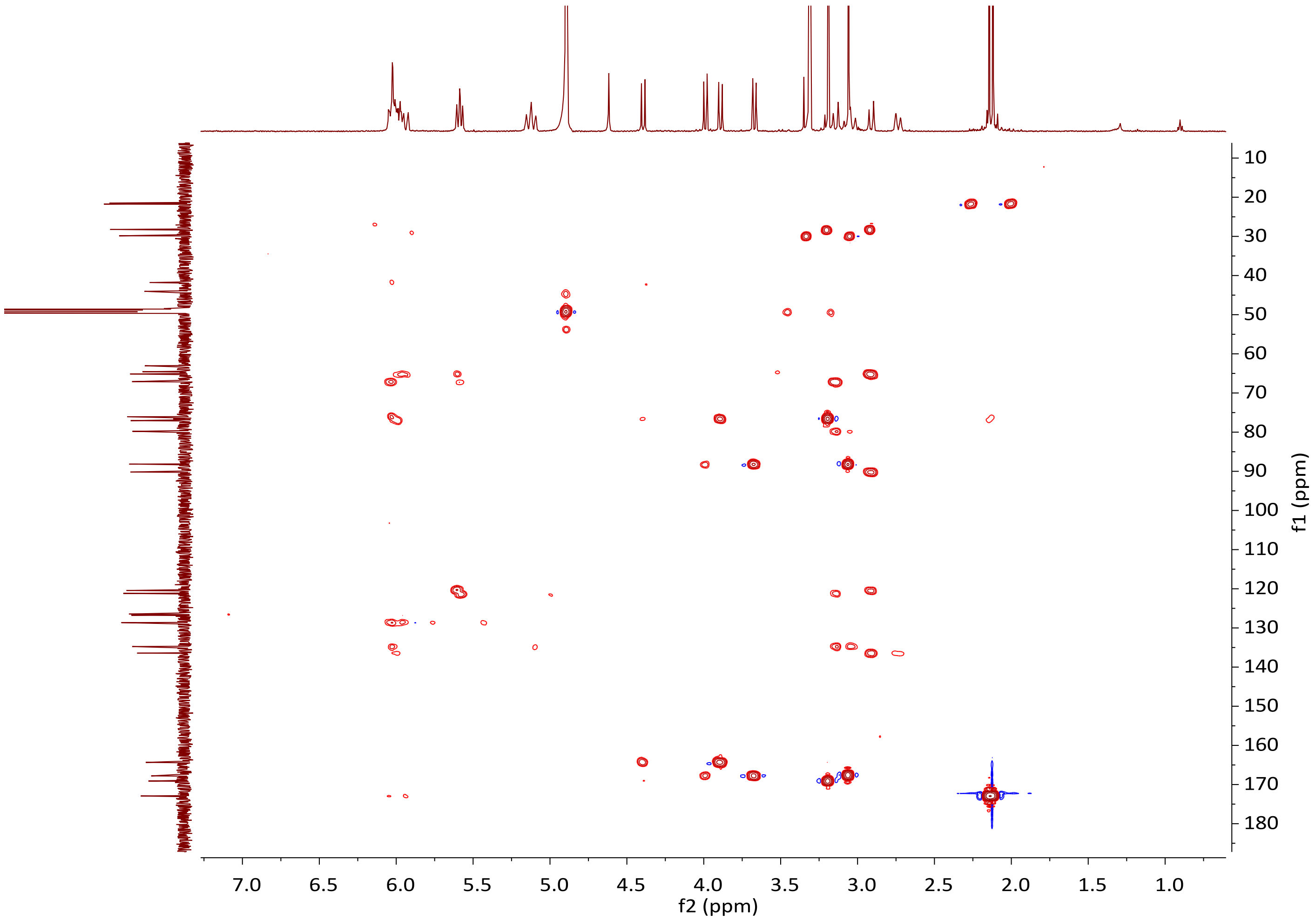

Figure S19. ^{1}H NMR spectrum of **3** in DMSO.

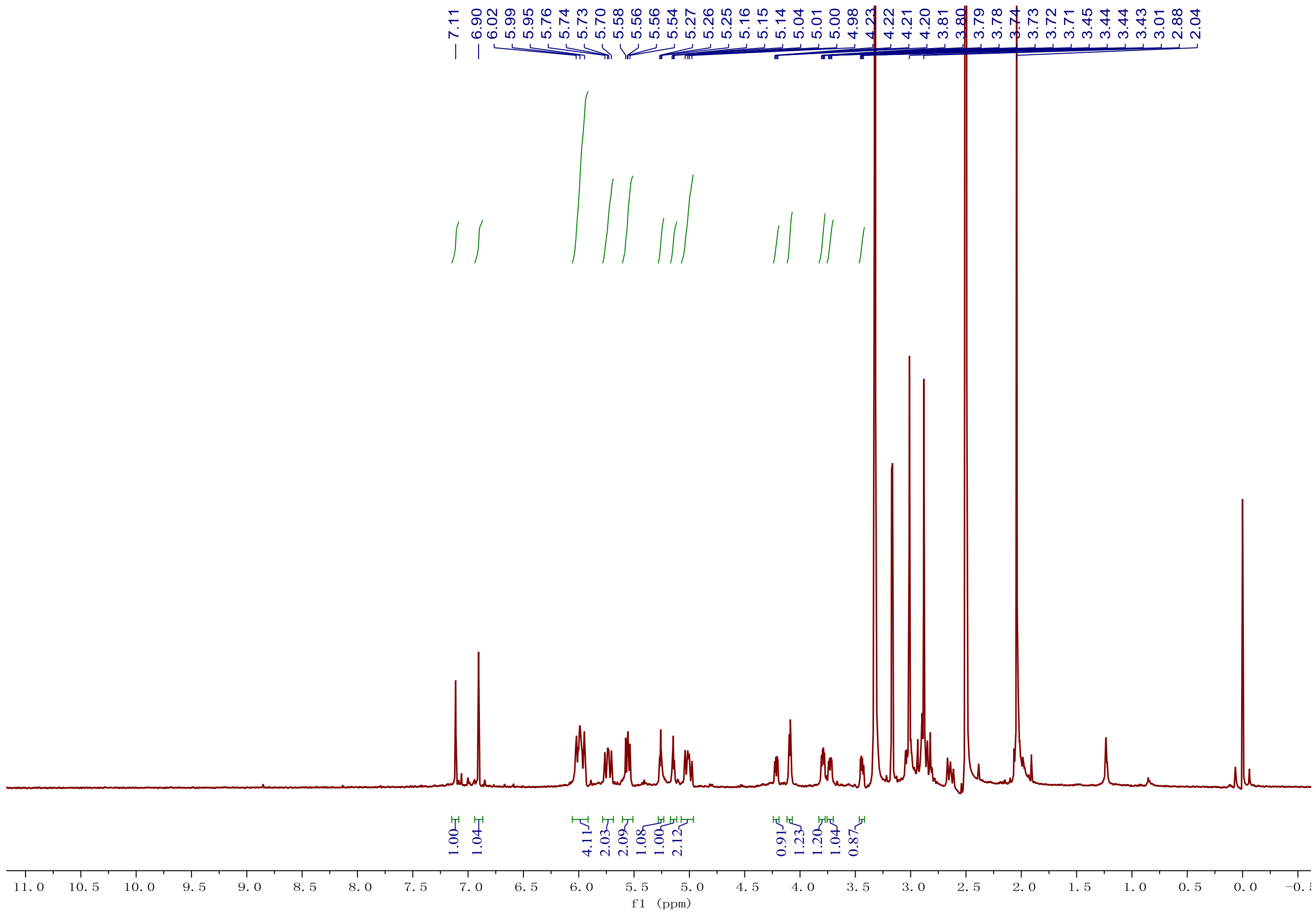

Figure S20. ^{13}C NMR spectrum of **3** in DMSO.

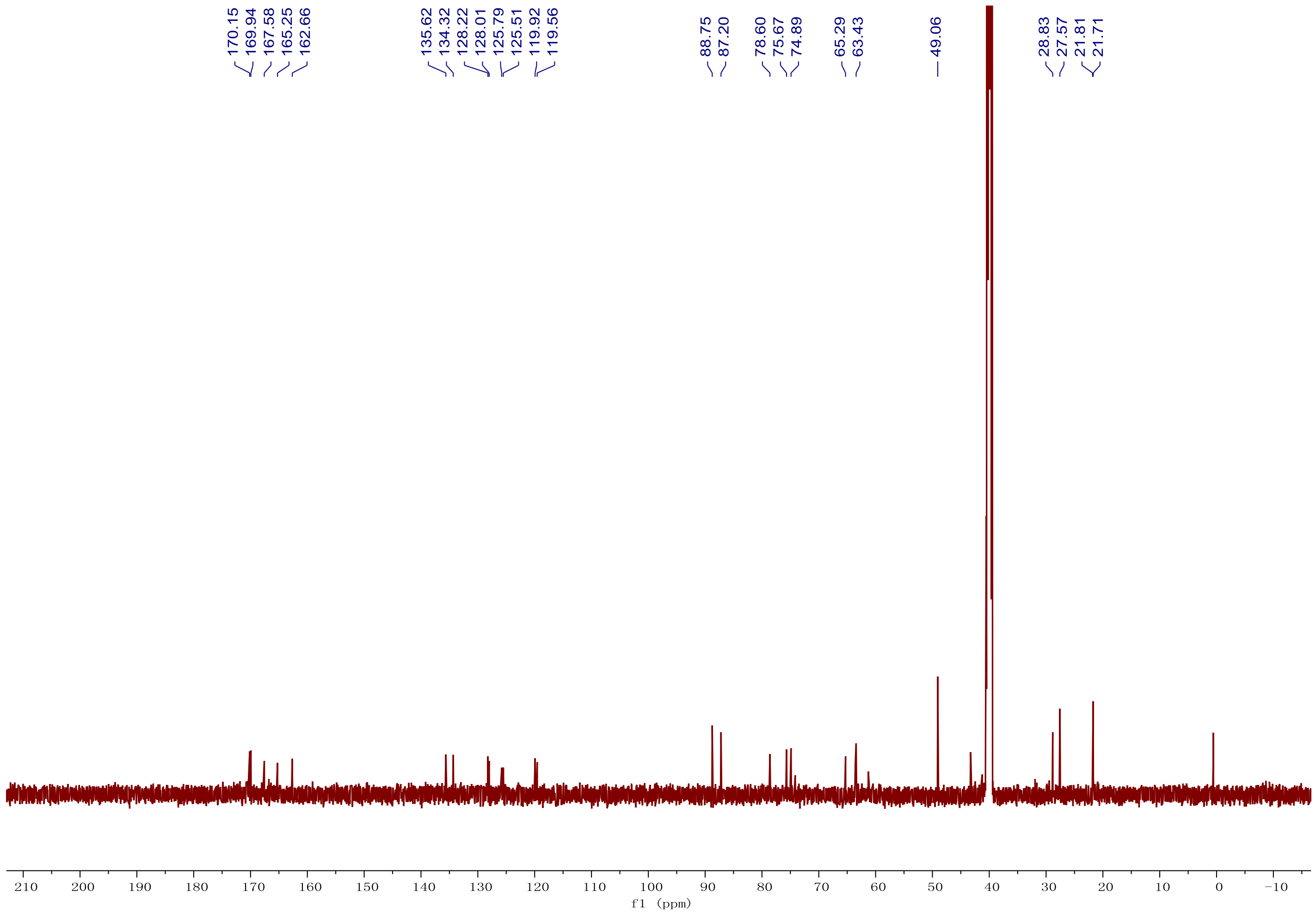

Figure S21. ^{1}H, ^{1}H-COSY spectrum of **3** in DMSO.

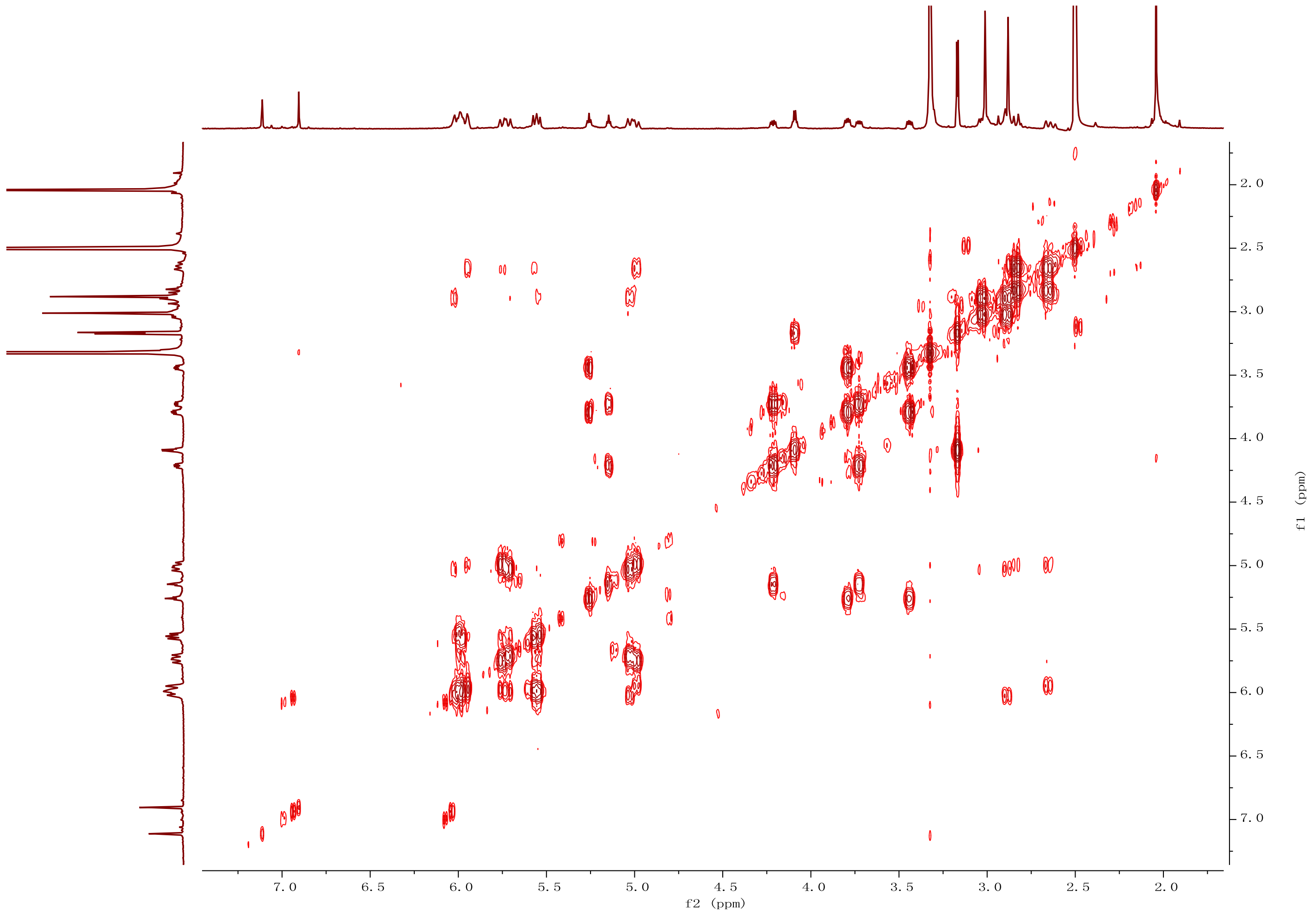

Figure S22. HSQC spectrum of **3** in DMSO.

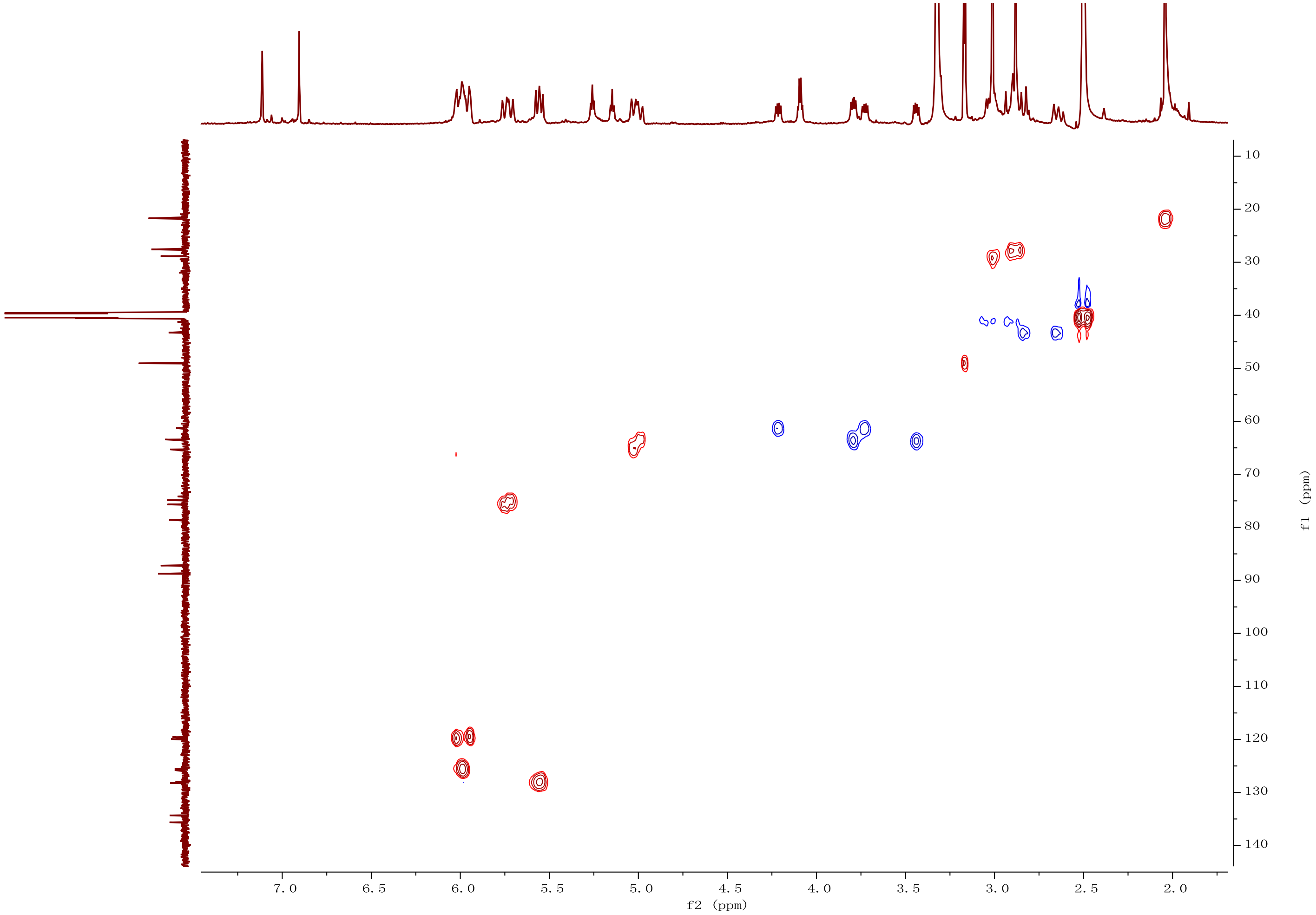

Figure S23. HMBC spectrum of **3** in DMSO.

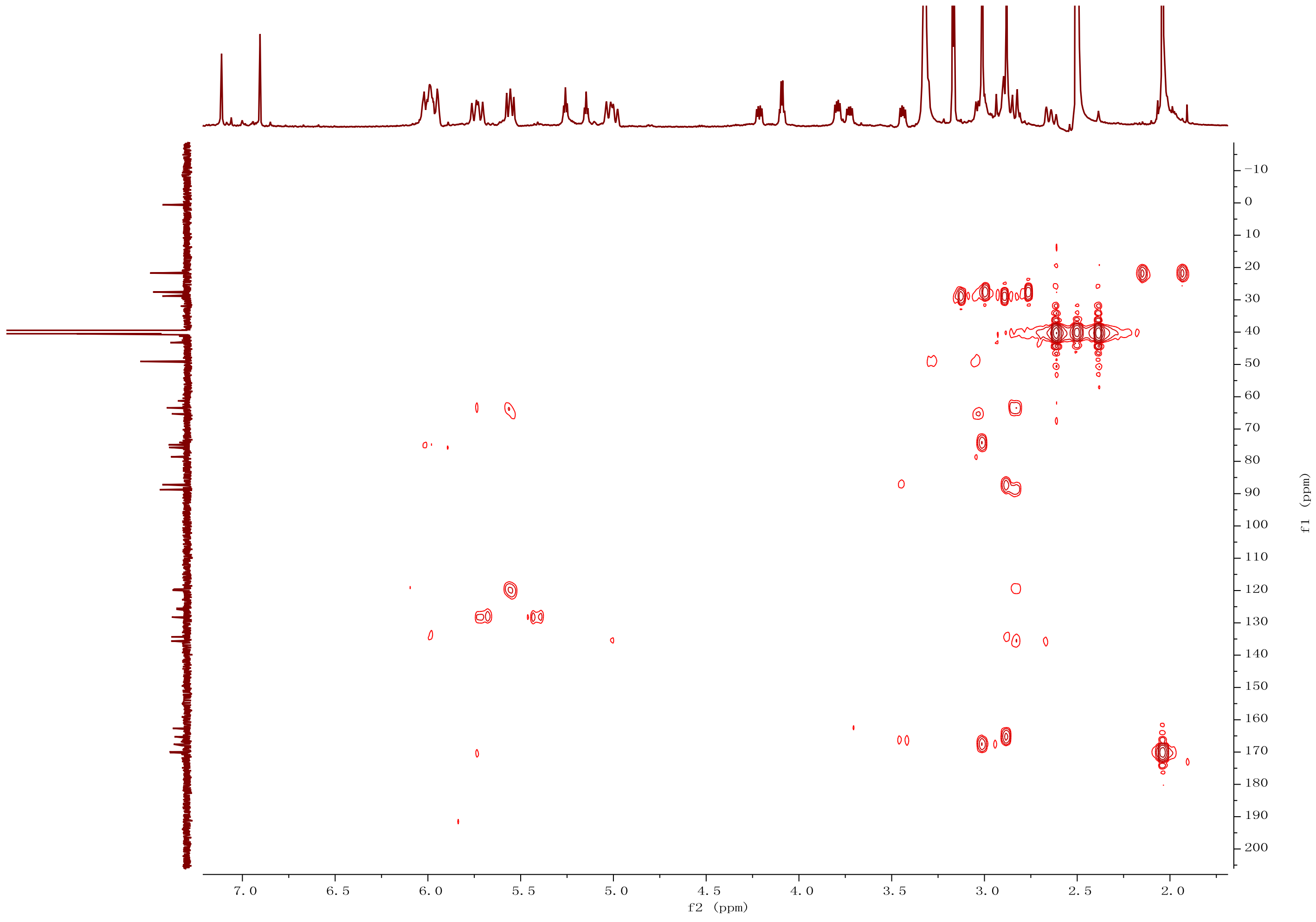

Figure S24. NOESY spectrum of **3** in DMSO.

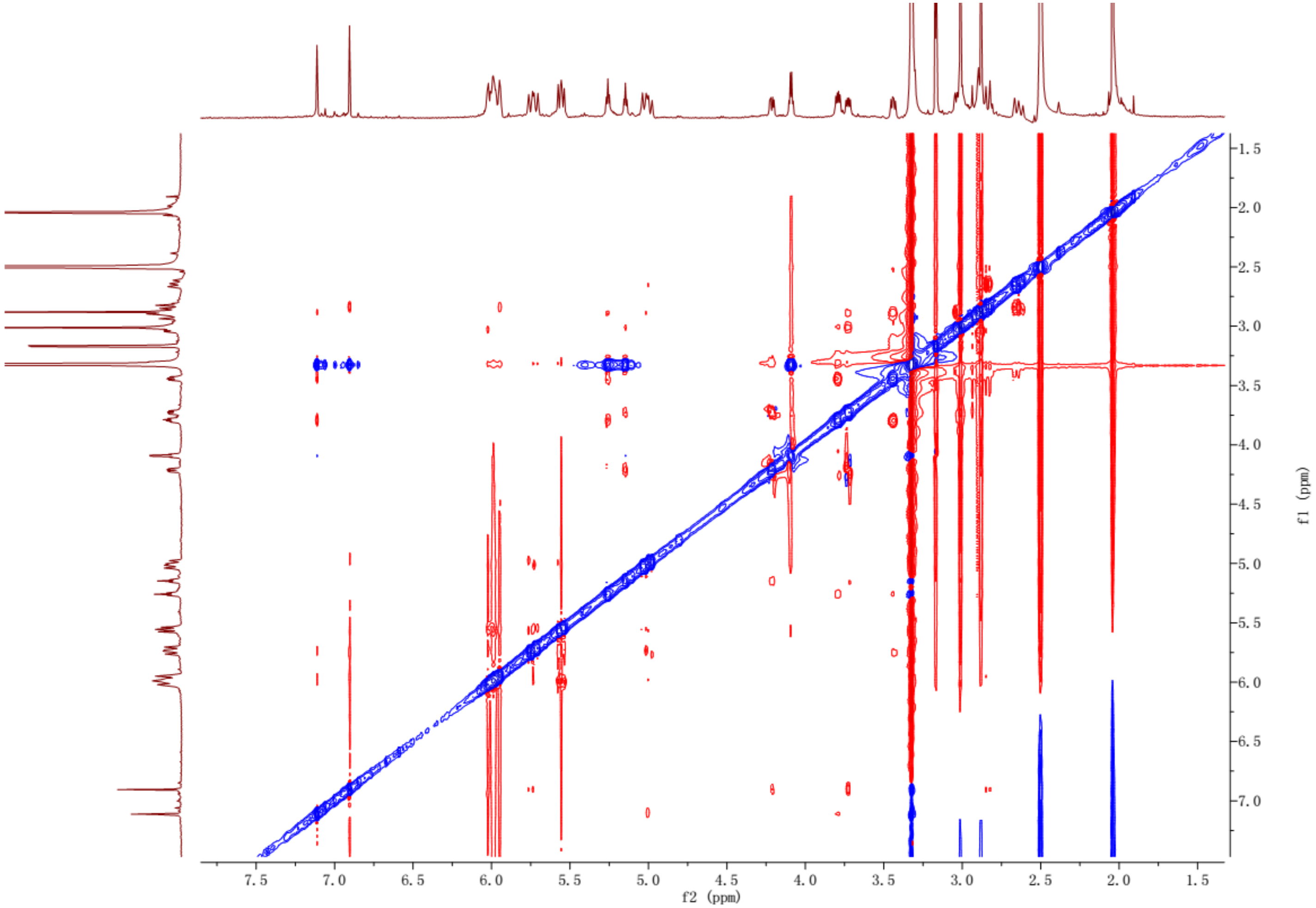

Figure S25. HRESIMS spectrum of **1**.

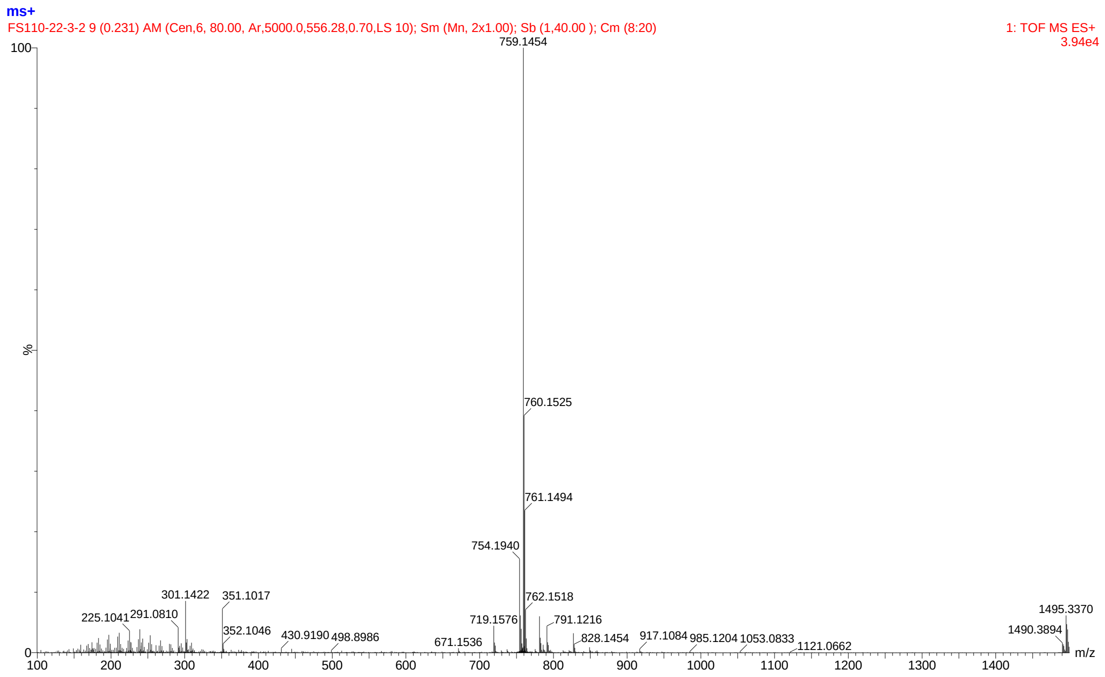

SPECTRUM - simulation :

m/z	Theo. Mass	Composition
759.1454	759.1440	$C_{31}H_{36}O_{11}N_4S_3Na$

Limits:

1) Charge: -1
2) Nitrogen-role: Do not use
3) Mass tolerance: 5 ppm
4) Element in use: ^{12}C(0~30), ^{1}H(0~60), ^{16}O(0~10)

Figure S26. HRESI MS spectrum of **2**.

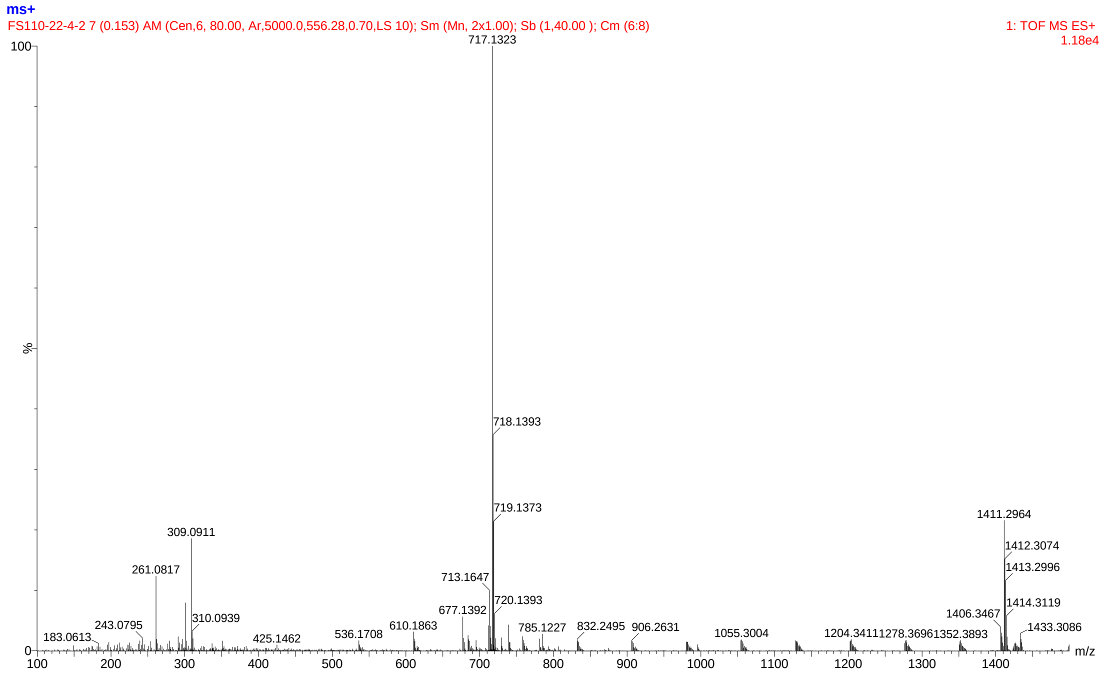

SPECTRUM - simulation :

m/z	Theo. Mass	Composition
717.1323	717.1335	$C_{29}H_{24}O_{10}N_4S_3Na$

Limits:

1) Charge: +1
2) Nitrogen-role: Do not use
3) Mass tolerance: 5 ppm
4) Element in use: $^{12}C(0\sim30)$, $^{1}H(0\sim60)$, $^{16}O(0\sim10)$, $^{35}Cl(0\sim1)$

Figure S27. HRESI TOF MS spectrum of **7**.

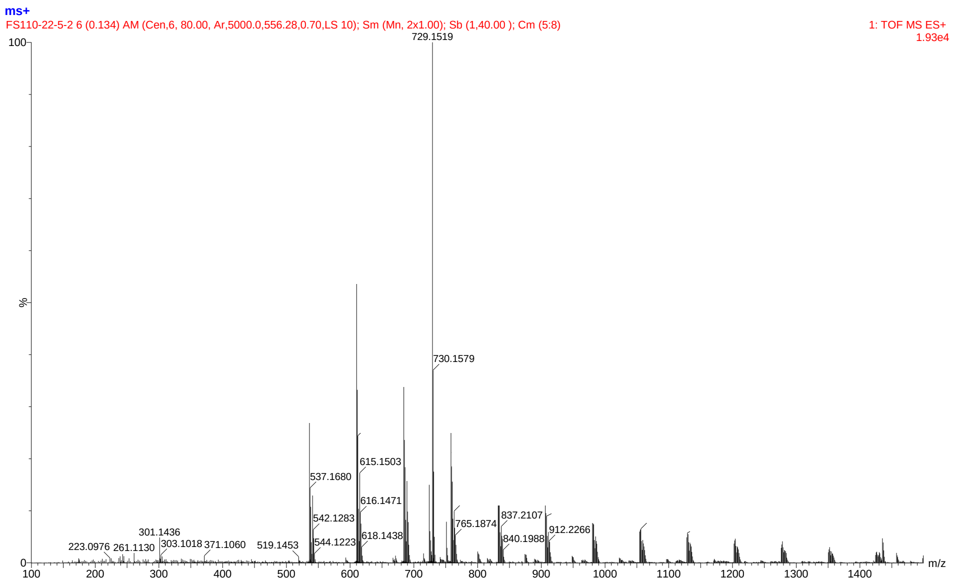

SPECTRUM - simulation :

m/z	Theo. Mass	Composition
729.1519	729.1512	$C_{30}H_{34}O_{12}N_4S_2Na$

Elemental Composition Report

1. Monoisotopic Mass
2. Elements Used: C: 0-40 H: 0-100 O: 0-20
3. Tolerance = 5.0 PPM

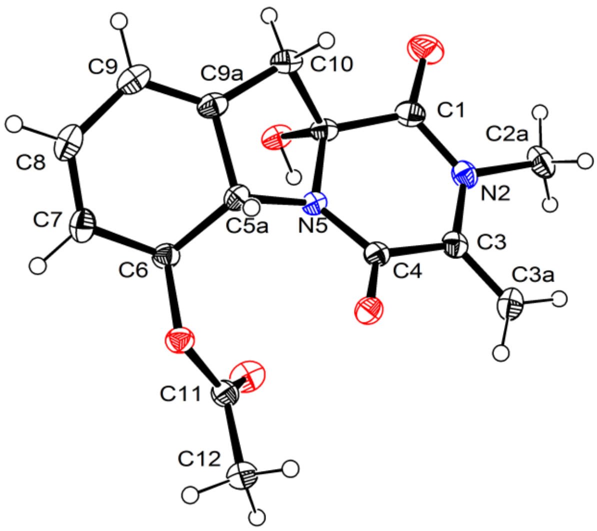

Figure S28. The crystal structure of **5**.

Table S1. ^{1}H and ^{13}C NMR data of **1−3**.

position	δ_H (*J* in Hz)	δ_C, *mult.*	δ_H (*J* in Hz)	δ_C, *mult.*	δ_H (*J* in Hz)	δ_C, *mult.*
		a		b		b
1		167.0, C		169.0, C		169.1, C
2						
3		74.4, C		78.2, C		76.6, C
4		162.1, C		164.2, C		164.3, C
5						
5a	5.00, d 14.2	63.0, CH	5.17, brd, 14.4	65.2, CH	5.13, m	65.1, CH
6	5.75, d (14.2)	75.3, CH	5.93, m	77.2, CH	5.93, m	77.1, CH
7	5.99, m	127.8, CH	5.60, m	128.8, CH	5.98, m	126.6, CH
8	5.58, t (10.3)	125.4, CH	5.99, m	126.6, CH	5.60, brd, 9.5	128.7, CH
9	5.95, m	119.1, CH	5.98, m	120.6, CH	5.96, m	120.4, CH
9a		135.5, C		136.3, C		136.4, C
10	2.85, d (16.6)	42.8, CH_2	2.94, d, 14.8	44.0, CH_2	2.91, d 14.7	44.0, CH_2
	2.67, d (16.6)		2.75, brd 14.8		2.74, m	
10a		88.4, C		90.1, C		90.2, C
11		169.8, C		172.4, C		172.9, C
12	2.0, s	21.2, CH_3	2.09, s	21.6, CH_3	2.14, s	21.8, CH_3
13	3.03, s	28.9, CH_3	3.18, s	30.1, CH_3	3.19, s	29.9, CH_3
14	4.33, dd 11.7 5.3	61.7, CH_2	4.40, d 11.6	64.0, C H_2	4.39, d 11.6	63.0, CH_2
	3.85, dd 11.7 6.3		3.99, d 11.6		3.89, d 11.6	
1′		165.0, C		167.1, C		167.6, C
2′						
3′		73.0, C		73.1, C		88.2, C
4′		163.2, C		167.8, C		167.8, C
5′						
5a′	5.06, d 14.3	65.4, CH	4.96, brd 13.6	70.7, CH	5.13, m	67.1, CH
6′	6.0, m	74.4, CH	4.86, brd 13.6	75.4, CH	6.04, m	76.1, CH
7′		127.4, CH	5.68, m	130.9, CH	5.98, m	126.4, CH
8′		125.7, CH	5.94, m	124.8, CH	5.58, brd, 9.5	128.7, CH
9′		120.0, CH	6.06, m	121.4, CH	6.02, m	121.2, CH
9a′		133.3, C		133.0, C		134.8, C
10′	3.41, d 16.4	38.8, CH_2	3.49, d 16.2	40.1, CH_2	3.14, d 16.1	41.8, CH_2
	2.86, d 16.4		3.08, d 16.2		3.03, m	
10a′		77.1, C		78.7, C		79.8, C
11′		169.8, C	-	-		172.9, C
12′	2.0, s	21.2, CH_3	-	-	2.12, s	21.5, CH_3
13′	2.96, s	28.5, CH_3	3.15, s	29.4, CH_3	3.06, s	28.2, CH_3
14′	4.05, dd 11.4 5.3	62.7, CH_2	4.34, d 11.7	63.6, CH_2	3.99, d 10.9	64.6, CH_2
	3.61, dd 11.3 6.6		3.92, d 11.7		3.67, d 10.9	

10a-OH	6.97, s		-	-	-	-
14-OH	5.28, t 5.8		-	-	-	-
S-CH_3	2.11, s	12.1, CH_3	2.27, s	13.8, CH_3	-	-
14′-OH	5.4, dd 6.6, 5.4		-	-	-	-

[a]Measured in dimethyl sulphoxide-d_6; [b]Measured in methanol-d_4.

Table S2. Energy analysis for the Conformers of **1a**.

compounds	Conformation	G (Hartree)	G (Kcal/mol)	ΔG (Kcal/mol)	Boltzmann Dist (%)
**1a**	**1a-1**	-3444.16452700	-2161223.229	0.644276052	22.23%
	**1a-2**	-3444.16555385	-2161223.873	0	66.00%
	**1a-3**	-3444.16392671	-2161222.852	1.021035069	11.77%

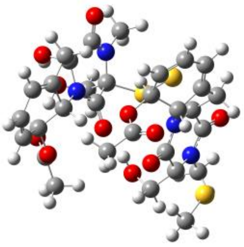

1a-1

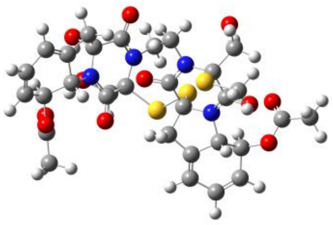

1a-2

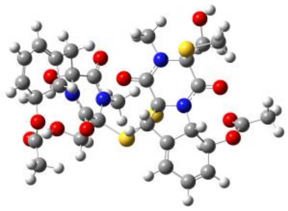

1a-3

Table S3. Energy analysis for the Conformers of **1b**.

compounds	Conformation	G (Hartree)	G (Kcal/mol)	ΔG (Kcal/mol)	Boltzmann Dist (%)
**1a**	**1a-1**	-3444.15263300	-2161223.229	0.644276052	22.23%
	**1a-2**	-3444.14852934	-2161223.873	0	66.00%

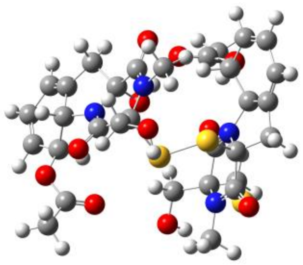

1b-1

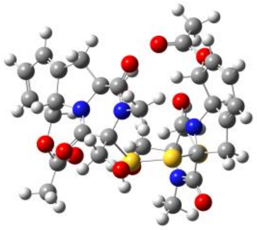

1b-2

Table S4. Energy analysis for the Conformers of **3a**.

compounds	Conformation	G (Hartree)	G (Kcal/mol)	ΔG (Kcal/mol)	Boltzmann Dist (%)
**3a**	**3a-1**	-3081.90018400	-1933901.303	0	62.89%
	**3a-2**	-3081.89466060	-1933897.837	3.465729892	0.18%
	**3a-3**	-3081.89968119	-1933900.987	0.315295107	36.39%

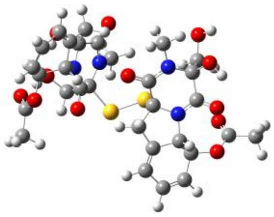

3a-1

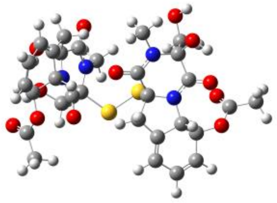

3a-2

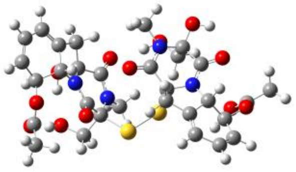

3a-3

Table S5. Energy analysis for the Conformers of **3b**.

compounds	Conformation	G (Hartree)	G (Kcal/mol)	ΔG (Kcal/mol)	Boltzmann Dist (%)
**3a**	**3b-1**	-3081.89794900	-1933899.901	0.098950932	45.83%
	**3b-2**	-3081.89810710	-1933900.000	0	54.16%

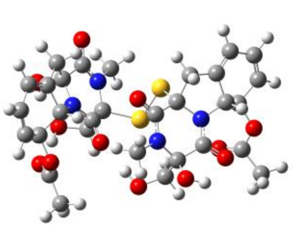

3b-1

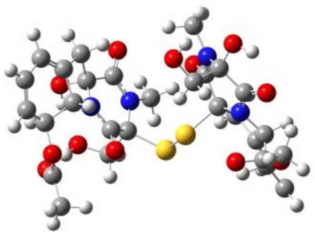

3b-2
